# ERR-activated GPR35 promotes immune infiltration level of macrophages in gastric cancer tissues

**DOI:** 10.1038/s41420-022-01238-4

**Published:** 2022-11-04

**Authors:** Chuanjun Shu, Can Wang, Saisai Chen, Xuan Huang, Jiahua Cui, Wenchao Li, Bin Xu

**Affiliations:** 1 Department of Bioinformatics, School of Biomedical Engineering and Informatics, Nanjing Medical University, Nanjing, 211166 China; 2 Department of Molecular Cell Biology & Toxicology, Center for Global Health, School of Public Health, Nanjing Medical University, Nanjing, 211166 China; 3 Department of Urology, Affiliated Zhongda Hospital of Southeast University, Nanjing, Jiangsu 210009 China; 4 Reproductive Medical Center, Jinling Hospital Affiliated to Medical School of Nanjing University, Nanjing, Jiangsu 210002 China

**Keywords:** Gastric cancer, Prognostic markers

## Abstract

Enhancer release and retargeting (ERR) events could activate disease-causing gene promoters for increasing the expression level of oncogenes. Meanwhile, class A orphan GPCRs (oGPCRs) are known as potential biomarkers or drug targets for various cancers, such as gastric cancer (GC). Hence, systemic investigation of ERR events for class A oGPCRs in GC could help to explore biomarkers for GC. In this study, ENCODE and GTEx eQTL data were utilized to define ERR events in GC. Only GPR35 was then detected that could be activated by ERR in GC based on these data and ChIP-seq. Then, activated GPR35 functional in GC cells were explored by flow cytometry, cell-based wound healing assay, Transwell migration assay, and M2 polarization of macrophages assay. Meanwhile, according to TCGA and GEO database, overall survival, immune-related gene expression, and immune cell infiltration level in different GPR35 expressions were calculated. Here, we found ERR event activate GPR35 results in GC cells proliferation and migration, and partly immune cells significance exhaustion (CD8 + T-cells and CD4 + memory T-cells) and/or infiltration (T-cells and macrophage). Meanwhile, high GRP35 level leads to a poor prognosis in GC patients, probably partly due to it promoting the immune infiltration level of macrophages and then inducing polarization of M2 macrophages. Notably, GPR35’s high expression in CTSB+ and CD68 + macrophage could be a genetic indicator for early warning of primary GC. Hence, our findings provide a novel activation approach for oGPCRs, and GPR35 could be determined as a new drugable receptor and early genetic indicator for GC.

## Introduction

Enhancer release and retargeting (ERR) event is a clinically critical paradigm that depends on genetic or epigenetic alterations neighbor to or within promoters, by which the defects of a non-disease-causing gene promoter cause the activation of alternative, disease-causing gene promoters in a shared chromatin domain [1]. One notable feature of ERR makes it applicable to explain the most abnormal expression of proteins in diverse tumors, i.e., it can cause a striking activation of alternative genes, such as oncogenes [1]. These alternative genes, when compared to normal conditions, are often increased in expression by two- to fivefold and sometimes by around dozens-fold.

Gastric cancer (GC) is one leading reason for global cancer morbidity and mortality, with particularly high incidence in Asia, Central America, and Eastern Europe [2]. In GC patients, there are many differentially expressed genes when tumors and matched tumor-adjacent (normal) tissues are compared [3]. In these differential expression genes, there are many belonging to G protein-coupled receptors (GPCRs), such as LGR4, LGR6, and GPR34 [4, 5]. GPCRs are the largest superfamily of signal transduction proteins, which regulate multiple physiological functions as well as tumor growth and metastasis [5–7]. Therefore, ERR event probably increases the expression level of cancer-related GPCRs and then activates their functions in GC tumor occurrence and development.

Currently established GPCR drug targets are widely utilized by distinct approved agents [7, 8]. Meanwhile, despite improvements in clinical diagnosis and therapeutic strategies, the prognosis of GC patients remains poor due to generally discovered too late, a high recurrence rate, and distant metastasis [9–11]. These present situations emphasize the necessity of expanding to new druggable receptors in order to develop novel medications for GC therapy. Importantly, the endogenous ligands for a large group of GPCRs have not yet been identified and are therefore known as orphan GPCRs (oGPCRs) [12]. Additionally, some stomach cancer-related GPCRs also belong to oGPCRs, such as GPR34 [4, 5]. However, growing evidence from animal studies, together with genome-wide association studies and post-mortem transcriptomic analysis in patients, pointed at many oGPCRs could act as potential biomarkers or pharmacological targets for distinct cancers [8, 12, 13]. Hence, oGPCRs could act as a good option for candidate indictors/drugs in early detection/therapy GC through a series of basic researches and clinical trials.

In this study, an oGPCRs i.e., GPR35 was found it not only is a prognosis-related gene in GC patients, but also has an ERR event. GPR35 was discovered in 1998, which has garnered interest as a potential therapeutic target through its association with many diseases, although its endogenous ligand or constitutive activation remains unknown. It is known to be highly expressed in lower intestine and colon cancers, in a variety of immune cells, including monocytes and a variety of dendritic cells, and in dorsal root ganglia [14, 15]. Meanwhile, the deletion of GPR35 selectively in the intestinal epithelium was sufficient to reduce tumor numbers [14]. However, GPR35 was found it was also highly expressed in stomach tissues and related to the development and immune infiltration for gastric cancer tumors in this study. GPR35 remains poorly characterized and has been slow to amass interest in gastric cancer therapy. Hence, we then observed that ERR event probably by increasing GPR35 expression level in CTSB+ and CD68 + macrophage and then promoting macrophage infiltration level and M2 polarization that leads to deterioration of the stomach, such as primary GC. Hence, GPR35 probably is a good druggable receptor and biomarker for GC. Its expression level in CTSB+ and CD68 + macrophage could act as an indicator for early warning of primary gastric cancer. Our results may provide new insight into the oGPCRs activation pathway, and GPR35 may benefit GC patients as a novel early indicator and a druggable receptor.

## Results

### GPR35 is activated by enhancer release and retargeting events in gastric cancer

**Fig. 1:**
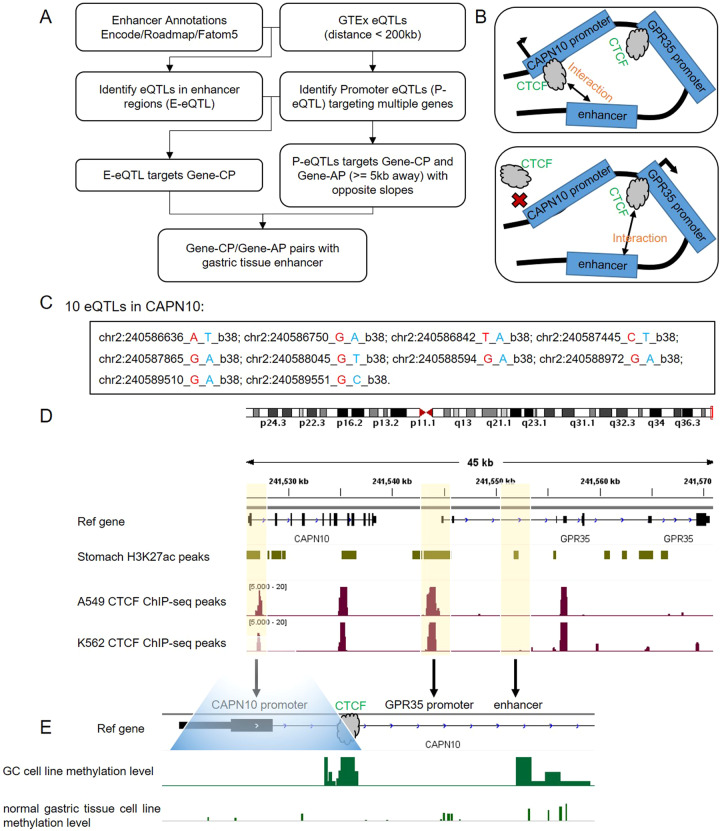
ERR events in the stomach. **A** Overview of workflow to identify ERR events. **B** Enhancer scanning model to interpret ERR events. CAPN10 and GPR35 were identified as ERR evens in the stomach. **C** All P-eQTLs of CAPN10. **D** CTCF ChIP-seq signals on CAPN10 and GPR35. **E** The DNA methylation levels of CAPN10 promoter in GA and normal gastric tissue cell lines.

Here, we used ENCODE and GTEx eQTL data to define ERR events in gastric cancer. The standard pipeline was shown in Fig. 1A. All eQTLs and their significant variant-gene were obtained from GTEx V8.0 [16]. All cis-eQTLs of the stomach in promoter regions, 3 kb upstream and 3 kb downstream of transcription start sites (TSSs) of target genes, were defined as promoter eQTLs (P-eQTLs). We refer to these target genes containing p-eQTLs as cognate promoters (gene-CP). Only P-eQTLs, which also act as eQTLs for a distal gene in their chromosomal neighborhood (±200 kb) in the stomach, were retained. These distal genes were referred to as gene-AP. Next, we used H3K27ac ChIP-seq data of the stomach from ENCODE project as enhancers. The enhancers containing cis-eQTLs, which target gene-CP were retained. Finally, the enhancers, gene-CP, and gene-AP were regarded as ERR events.

Totally, we identified 853 ERR events in the stomach (Supplementary Table S1). We found that a class A orphan GPCR GPR35 and its neighbor gene CAPN10 were ERR events in the stomach (Fig. 1B and Supplementary table S2). In this case, the enhancer preferentially interacted with the promoter of CAPN10 by the chromatin looping extrusion model. When the CTCF-binding region in the promoter of CAPN10 was mutated, GPR35 can be activated by the enhancer due to chromatin looping extrusion (Fig. 1B). In total, we found that 10 eQTLs were located in the promoters of CAPN10 (Fig. 1C). We used H3K27ac ChIP-seq data to define enhancers in the stomach. We found that the promoters of CAPN10 and GPR35, and enhancers contain CTCF ChIP-seq signals in many cell lines (Fig. 1D), which indicates that these regions conserved contain CTCF binding. The cohesin proteins can be stalled by CTCF. In the normal stomach tissue, cohesin proteins were stalled at the enhancer and promoter of CAPN10 due to the property of CTCF. In gastric cancer, the DNA methylation levels of the promoter of CAPN10 became much higher, leading to loss of CTCF binding (Fig. 1E and supplementary Fig. S1). In this case, cohesin proteins were stalled at the enhancer and the promoter of GPR35, leading to activating GPR35.

**Fig. 2:**
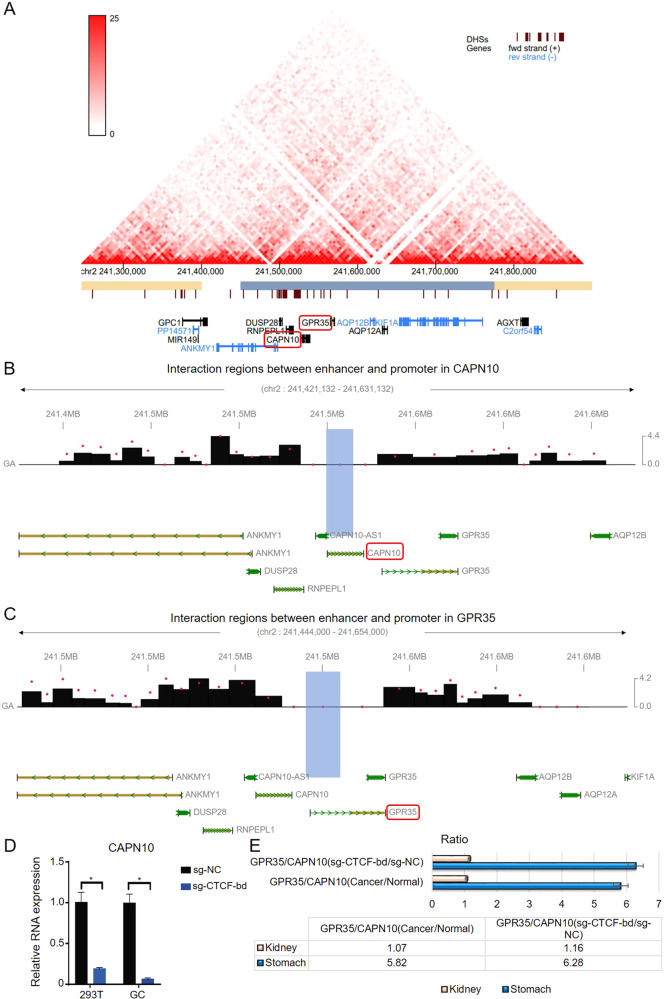
ERR event for orphan GPR35 in GC tumors. **A** GPR35 and CAPN10 were located in the same TAD. **B** Interaction between the enhancer and promoter of CAPN10. **C** Interaction between the enhancer and promoter of GPR35. **D** Knocked out CTCF-bd (CTCF-binding domain) influences CAPN10 expression level. **E** Compare kidney cancer, ERR-activated GPR35 expression in gastric cancer and cell.

We used Hi-C data to check the chromatin interaction in the ERR event. From Fig. 2A, it can be seen that GPR35 and CAPN10 were located in the same topological associated domain (TAD), indicating the enhancer scan model is available in this case. We further adopted capture Hi-C data of gastric tissue from the 3Div database (http://www.3div.kr/). It can be seen that the enhancer frequently interacts with CAPN10 in the gastric tissue (Fig. 2B, C). Meanwhile, we found ERR event between GPR35 and CAPN10 was not found in kidney tissue (Supplementary Table S3). Hence, we compared GPR35 and CAPN10 expression fluctuation in kidney and gastric cancer tissues, but also their relationship in kidney cell and gastric cancer cells when knocked out CTCF-bd (binding domain) for CAPN10. Then, we found that knock-out CTCF-bd could result in a drastic decrease in the expression of CAPN10 in both two type cells (293 T and MKN-27) (Fig. 2D). Furthermore, compared to kidney cancer (Pan-kidney cohort, KIPAN), ERR event in gastric cancer resulted in a higher ratio between GPR35 and CAPN10 (Fig. 2E). This phenomenon in corresponding cells was also detected when knocked out CTCF-bd in CAPN10 (Fig. 2E). These results indicated that ERR event could result in low expression for CAPN10 and high expression for GPR35 in gastric cancer.

Taken together, we found that ERR events frequently happen in the gastric tissue. We also proved that orphan GPR35 is potentially activated by ERR events in gastric cancer.

### ERR event for GPR35 is a critical adverse factor for GC prognosis

**Fig. 3:**
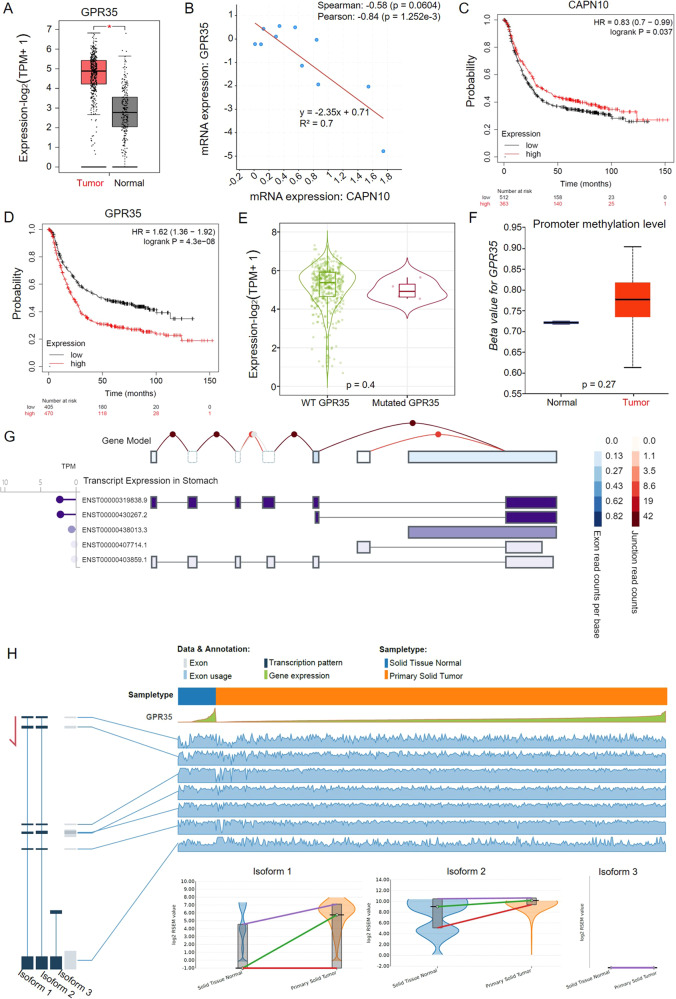
High expression for GPR35 represents the bad prognosis for GC patients. **A** GPR35 expression level in GC tumor and matched normal tissues. **B** Relation between mRNA expression levels for GPR35 and CAPN10. **C**, **D** Kaplan–Meier curves depicting OS according to the expression patterns of CAPN10 (**C**) and GPR35 (**D**) in the GC cohort. *P* values were calculated with the log-rank test. **E** Differential GPR35 variations expression level in GC. *P* value was computed with the Mann–Whitney test. **F** Promoter methylation level of GPR35 in GC and normal tissues. *P* value was computed with the *t*-test. **G** Transcript expression of GPR35 in the stomach. **H** Alternative splicing events for GPR35 in GC and its corresponding normal solid tissues.

To explore the significance of GPR35 in GC patients, differential expression and survival analysis were first performed based on TCGA and GEO database. Then, GPR35 was found that there is an obvious significant high expression in GC tumors, when compared to its level in tumor-adjacent (normal) tissues (Fig. 3A). Meanwhile, a significant negative correlation for mRNA expressions was found between GPR35 and CAPN10 based on the lasted study GC data (Pearson correlation, *r* = −0.84, *p* = 1.25e-3, Fig. 3B), indicating that the ERR event probably not only active GPR35 but also partly contribute to decreasing the expression level of CAPN10 in GC tumors.

According to the GEO database (GSE14210, GSE15459, GSE51105, GSE22377, GSE62254, and GSE29272), the Kaplan–Maier Survival plot was utilized to show overall survival for CAPN10 and GPR35 (Fig. 3C, D). The significance HR (hazard ratio) for CAPN10 and GPR35 is 0.83 (*p* = 0.03) and 1.62 (*p* = 4.30e-08), respectively. The survival plots and HR values suggested that GPR35 is a promote cancer gene, nevertheless, CAPN10 is a suppressor cancer gene in GC. It indicated that high expression levels of GPR35 predicted poor prognostic in GC patients. Furthermore, the mutation frequency for GPR35 in GC tumors is 2.7% which is obtained from cbioportal database (www.cbioportal.org) (Supplementary Fig. S2A, D). However, there was no significant difference in expression between wild type (WT) and mutated GPR35 in GC tumors (*p* > 0.05) (Fig. 3E). Additionally, as shown in Fig. 3F, the promoter methylation level of GPR35 has no obvious difference between tumor-adjacent (normal) and tumor tissues (*p* > 0.05). Meanwhile, based on GEO and TCGA data, alternative splicing events in gastric normal and cancer tissues were no significance different (Fig. 3G, H and Supplementary Fig. S2C). These results suggested that ERR event is probably the main reason for the high expression of GPR35 in GC tumors and then leads to poor prognosis of gastric cancer.

**Fig. 4:**
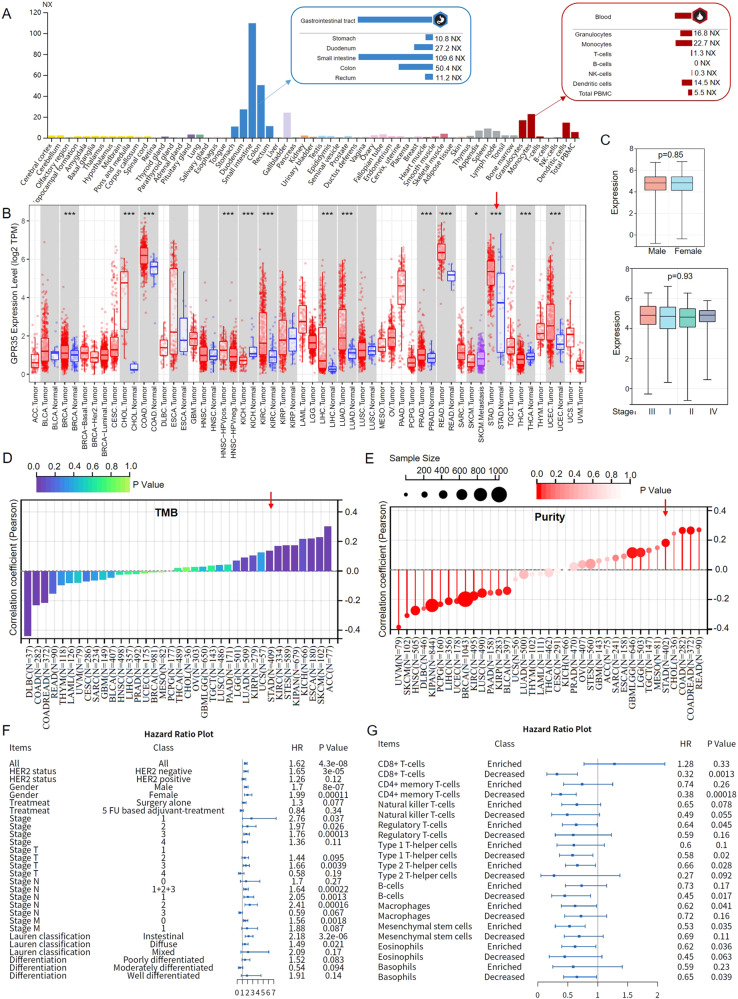
GPR35 promotes cancer development and immune cells decreased in GC tumors. **A** Tissues and blood specificity of GPR35 in human. NX represents normalized expression. The consensus RNA data is based on normalized expression (NX) data from three different sources: Human Protein Atlas (HPA), GTEx and FANTOM5 project. **B** GPR35 expression landscape in cancers and corresponding normal tissues. **C** GPR35 expression level in different gender and stages for GC. The landscape of TMB (**D**) and purity (**E**) for GPR35 in cancers. **F** HR (hazard ratio) values for GPR35 in different clinical groups. **G** HR values for GPR35 in enriched or decreased immune cells. *P* values were calculated with the log-rank test.

Since GPR35 is an orphan GPCR of which function remains unknown, GPR35 in tissues and blood specificity were systemically investigated in the human protein atlas (www.proteinatlas.org). As shown in Fig. 4A, GPR35 is enriched in the gastrointestinal tract (such as stomach, duodenum, small intestine, colon, and rectum) and some immune cells in the blood, i.e., monocytes, granulocytes, and dendritic cells. Meanwhile, we found that GPR35 not only has high expression in intestinal tumors (such as COAD and READ), but also in GC (Fig. 4B). However, many researches focus on GPR35 functions in the intestine, not stomach cancer recently. Therefore, in this study, we focus on the function of GPR35 in gastric cancer and their tumor microenvironment. Then, based on the TCGA database, we found GPR35 high expression in male and female, and it also had high expression in each stage for GC (Fig. 4C). Meanwhile, GPR35 were found to have a significant positive relationship with tumor mutational burden (TMB) and tumor purity in GC patients (Fig. 4D, E). These results indicated that GPR35 probably widely influences the immunotherapy effect and tumor microenvironment in GC patients with different clinical features.

According to previous downloaded GEO data in this study, high GPR35 level was associated with worse survival in almost all groups, such as groups for HER2 negative, HER2 positive, male, female, diverse treatments, TNM stages, Lauren classification, and diverse differentiation (Fig. 4F). There were no clinical characteristics with a significance HR which is small than 1. Moreover, the HR values for GPR35 in groups for TNM stages, Lauren classification, and diverse differentiation indicated that GPR35 plays a critical factor in the cancer development of GC (Fig. 4F). Hence, GPR35 could act as a genetic indicator of primary stomach cancer and a good drug target for almost all clinical types of GC patients.

Furthermore, since GPR35 was enriched in immune cells of the blood, the GPR35 function in the tumor immune microenvironment was explored. Its roles in enriched and decreased immune cells, i.e., CD8 + T-cells, CD4 + memory T-cells, nature killer T-cells, regulatory T-cells, type-1/2 T-helper cells, B-cells, macrophage, mesenchymal stem cells, eosinophils, and basophils, were systemically investigated. We found that GPR35 could effectively promote many types of immune cells decreased, especially CD8 + T-cells, CD4 + memory T-cells, and B-cells (Fig. 4G). However, GPR35 only effectively promotes a few types of immune cells enriched, i.e., regulatory T-cells, type-2 T-helper cells, macrophage, and mesenchymal stem cells (Fig. 4G). These results indicated that GPR35 probably promotes some immune cell exhaustion (such as CD8 + T-cells and CD4 + memory T-cells) and infiltration levels (such as T-cells and macrophage) in GC tumors.

### GPR35 enhances the proliferation and migration of gastric cancer cells

**Fig. 5:**
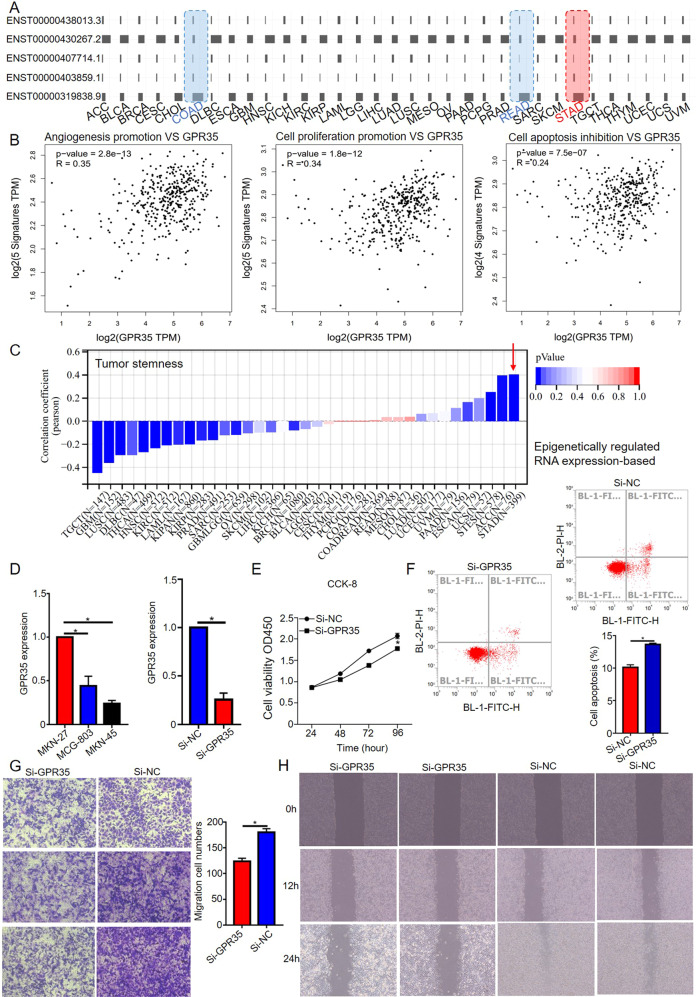
GPR35 promotes cell proliferation and migration for GC. **A** Landscape for transcript expression of GPR35 in pan-cancer. **B** The correlation between signature gene expression (angiogenesis, cell proliferation, and apoptosis) and GPR35 expression in GC. **C** Correlation coefficient between tumor stemness and GPR35 for different cancers. **D** GPR35 expression level in different GC cells. **E** Cell proliferation ability decreased after infected si-GPR35 (24 h). **F** si-GPR35 promotes cell apoptosis for MKN-27. The representative images and quantitative data of Transwell assays (**G**) and wound healing assays (**H**) of MKN-27 cells after different treatments, respectively.

Since GPR35 could enhance proliferation and migration in intestine cancer, GPR35 in GC cells were inferred to have the same effect in GC cells based on it has the same transcript expression model in COAD, READ, and STAD (Fig. 5A). Then, GPR35 functions in GC cells were systemic investigated based on bioinformatics and biology experiments analyze. Firstly, the GPR35 expression level was positively correlated with signature genes expression level for angiogenesis promotion (VEGF, CXCL1, TGFA, ANG, and Dll4), cell proliferation (Myc, PTEN, AKT, HSP90, and GSK3B) and cell apoptosis inhibition (BCL-xl, Bcl-W, Mcl-1, and 14-3-3) in GC (Fig. 5B). Meanwhile, the GPR35 expression level was positively related with RNA modifications genes expression level, such as m1A, m5C, and m6A (Supplementary Fig. S3A). Since RNA modification writers, readers and erasers can have either promoting or inhibitory effects on the hallmarks of cancer, the GPR35 expression level was then further explored the relationship with these signature genes’ expression in GC tissues. Then, we found GPR35 expression level has a positive relationship with promoting effects on hallmarks of cancer, especially escape apoptosis signatures (YTHDF2, PUS10, DCK1, ADAR1, and TUT1, *r* = 0.35, *p* = 1.91e-13) and achieve replicative immortality signatures (DKC1 and ADAR1, *r* = 0.32, *p* = 6.30e-13) (Supplementary Fig. S3B). Furthermore, for GC, the GPR35 expression level has a significant positive relative to the stemness of cancer cells which is a key feature for cancer progression and, in many cases, the source of cancer cell survival (Fig. 5C and Supplementary Fig. S4A). It also positive relative to signature genes expression which have high mutation frequency in GC tissues (PTEN, TP53, EGFR, RB1, and PKHD1; *r* = 0.2; *p* = 6.50e-05) (Supplementary Fig. S4B). Based on the TCGA database, these bioinformatics analysis results suggested that GPR35 has promoting effects on the hallmarks of cancer, such as escape apoptosis, achieve replicative immortality, acquire metastatic potential, and evade the immune response.

To verify our investigated results for bioinformatics analysis, cell viability assays, flow cytometer, Transwell assays, and wound healing assays were performed. Then, GPR35 were found high expression in GC cells, especially MKN-27 (Fig. 5D). Hence, MKN-27 was utilized to explore GPR35 functions in GC. Firstly, si-GPR35 were found that it could effectively reduce the expression level of GPR35 in MKN-27 (Fig. 5D). Based on the results of cell viability assays and flow cytometer, infected si-GPR35 (5 ul, 1.2 ug) in MKN-27 could result in lower cell viability and higher cell apoptosis ratio for cells when compared to control group cells (Fig. 5E, F). Furthermore, results of Transwell and wound healing assays indicated that GPR35 could promote cell migration for GC (Fig. 5G, H). Taken together, we found that GPR35 is an oncogene in GC, which could promote cancer cell proliferation and migration.

### GPR35 promotes immune infiltration level of macrophage and M2 macrophage polarization in GC tumors

To further explore the role of GPR35 in tumor immunity, we calculated the expression correlation of GPR35 with biomarkers of immune cells in STAD (stomach adenocarcinoma, i.e., GC) using the GEPIA database. As listed in Table 1, GPR35 was significantly negatively correlated with biomarkers for B-cells (CD19 and CD79A), CD8 + T-cells (CD8A and CD8B), CD4 + T-cells (CD4), M1 macrophage (CD86 and TLR4), neutrophil (ITGAM and CCR7), and dendritic cell (HLA-DPB1, CD1C, and NRP1) in GC. These results indicated high GPR35 levels could result in promoting many types of immune cell exhaustion in tumor tissues. In addition, GPR35 was significantly positively correlated with a biomarker for M2 macrophage (PPARG) in GC (Table 1). Taken together, these findings partially support that GPR35 is linked to immune cell exhaustion and infiltration (such as M2 macrophage polarization).

**Table 1:** Correlation analysis between GPR35 and biomarkers of immune cells in GC.

Immune cell	Biomarker	*R* value	*p* value
B cell	CD19	−0.18	0.00032**
	CD79A	−0.19	0.000093**
CD8 + T cell	CD8A	−0.13	0.011*
	CD8B	−0.12	0.012*
CD4 + T cell	CD4	−0.13	0.011*
M1 macrophage	CD86	−0.15	1.9E-03**
	IRF5	0.013	0.8
	TLR4	−0.13	7.7E-03**
M2 macrophage	PPARG	0.26	1.4E-07**
	Fizz1	−0.031	0.54
	IL-10	−0.091	0.067
Neutrophil	CEACAM8	−0.023	0.64
	ITGAM	−0.14	0.0052**
	CCR7	−0.16	0.0011**
Dendritic cell	HLA-DPB1	−0.14	0.0047**
	HLA-DQB1	−0.034	0.5
	HLA-DRA	−0.11	0.031*
	HLA-DPA1	−0.12	0.017*
	CD1C	−0.22	6.5E-06**
	NRP1	−0.16	0.0011**
	ITGAX	−0.032	0.52

^*^represents *p* < 0.05; ** represents *p* < 0.01.

**Fig. 6:**
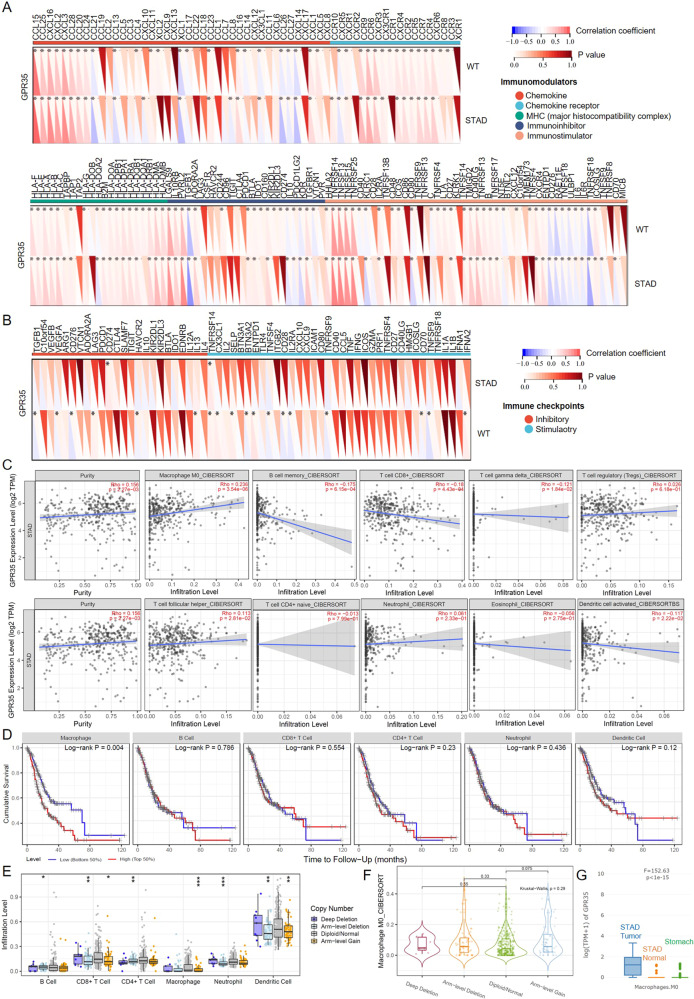
GPR35 links infiltration of immune cells in GC tumors. **A**, **B** Correlation coefficient between signature genes (immunomodulators (**A**) and immune checkpoints (**B**)) and GPR35 expression level. **C** Relation between GPR35 expression level and infiltration level of various immune cells. **D** Cumulative survival for different infiltration levels of immune cells in GC patients. **E** The infiltration level of various immune cells under different copy numbers of GPR35 in GC. *, **, and *** represents *p* < 0.05, *p* < 0.01, and *p* < 0.001, respectively. **F** The infiltration level of macrophage M0 under different copy numbers of GPR35 in GC. *P* values were calculated with the Kruskal–Wallis test. **G** GPR35 expression level in stomach normal tissues, tumor-adjacent tissues, and STAD (GC) tumors. *P* value was calculated with the *F*-test.

To explore the influence of GPR35 expression level in infiltration levels for diverse immune cells, the relationship between GPR35 and immunoregulatory/ immune checkpoint genes were first calculated. The results indicated that GPR35 could influence the effect of immunotherapy, such as PD-L1 (CD274) immunotherapy (Fig. 6A, B). This phenomenon is probably due to GPR35 influencing infiltration levels for immune cells. Then, the timer and cibersoft algorithm were performed for the mRNA expression matrix for GC tumors. We found that GPR35 expression level has a relationship with infiltration levels for most immune cells (Fig. 6C). As shown in Fig. 5C, GPR35 expression level has a significant negative relation with three types of immune cells, i.e., B cell memory (*r* = −0.17), T cell CD8 + (*r* = −0.18), T cell gamma delta (*r* = −0.12), and dendritic cell activated (*r* = −0.11). However, it has a significant positive relation with types of immune cells, i.e., Macrophage M0 (*r* = 0.23), T cell regulatory (*r* = 0.23), and T cell follicular helper (Fig. 6C and Supplementary Fig. S5). Then, infiltration levels for immune cells were further utilized to research their relation to cumulative survival for GC patients. We found that only macrophage infiltration level has a significant effect on cumulative survival for GC patients (*p* < 0.05) (Fig. 6D). A higher infiltration level of macrophage means a worse cumulative survival in the GC patients (Fig. 6D). Meanwhile, many significant changes of immune cell infiltration level under various copy number for GPR35 in GC were observed (Fig. 6E). However, there was only arm-level gain style has a small fluctuation on macrophage infiltration level (Fig. 6E). Furthermore, no significant changes of macrophage M0 infiltration level under various copy number for GPR35 in GC (Fig. 6F). Furthermore, according to the database of GTEx (genotype-tissue expression) and TCGA, GPR35 was found that its expression level among macrophage M0 in tumor tissues is far more than that in normal stomach tissues and tumor-adjacent tissues (Fig. 6G). Hence, ERR event increasing GPR35 expression level probably leads to a bad prognostic for GC patients by promoting macrophage infiltration level.

**Fig. 7:**
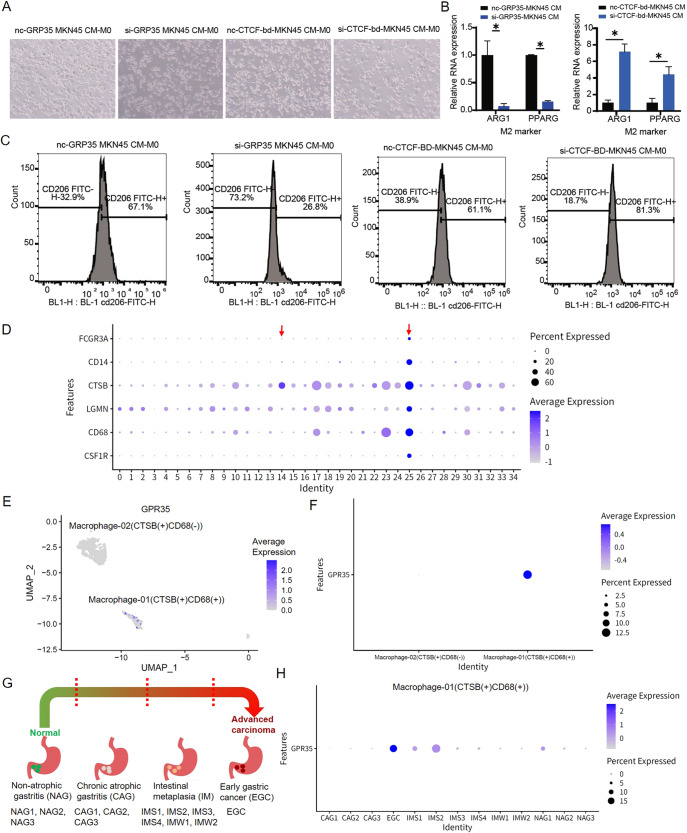
GPR35 functions in M2 polarization and gastric cancer development. **A** The representative images of M0 macrophages morphological change (from oval cells to antennal, spindle-shaped adherent cells) for different treated groups, i.e., si-GPR35, si-CTCF-bd of CAPN10, and negative control. **B** Quantitative data of M2 markers in different treated groups. **C** Relative positive rate for CD206 in different treated groups. **D**–**H** GPR35 levels in different cluster macrophages in gastric premalignant lesions and early gastric cancer. **D** Average expression for biomarkers of macrophage in clusters for cells of various gastric disease tissues. **E**–**F** UMAP (**E**) and bubble (**F**) diagram for GPR35 expression level in macrophage-01/02. **G** Sample composition for scRNA-seq. **H** GPR35 expression level for CTSB+ and CD68 + macrophage in various samples.

To investigate GPR35 functions in the process for M2 macrophage polarization, which is a key regulator of the link between inflammation and cancer, different treated MKN45 cells (GC cells) were utilized to culture M0 macrophages. Then, as shown in Fig. 7A, inhibit GPR35 expression and knock out CTCF-bd of CAPN10 in GC cells could result in less and more M0 macrophage antennas, respectively, when compared to negative treated groups. Meanwhile, relative RNA expressions for M2 marker genes, i.e., ARG1 and PPARG, in different treated groups were indicated that knock out CTCF-bd could result in M2 macrophage polarization (Fig. 7B). However, inhibiting GPR35 expression in GC cells could lead to suppressing M2 macrophage polarization (Fig. 7B). Furthermore, positive rate of surface molecule CD206 in M2 macrophages were detected by flow cytometry (Fig. 7C). Then, we found that GPR35 expression inhibited is along with the decrease of the proportion of CD206 up to 2.5-fold (from 67.1 to 26.8%) (Fig. 7C). Meanwhile, we also found that knock CTCF-db of CAPN10 leads to the increase of the proportion of CD206 up to 1.3-fold (from 61.1 to 81.3%) (Fig. 7C). Moreover, the correlation coefficient values between GPR35 and tumor-associated macrophage markers (VEGF and CD47) were all positive (*p* < 0.05) (Supplementary Fig. S6A). Taken together, these results suggested that GPR35 could promote M2 macrophage polarization in GC tissues.

### GPR35 level in CTSB+ and CD68 + macrophage could be an indicator of primary GC

To explore the molecular features of macrophage which is affected by GPR35, we downloaded single-cell RNA sequencing data in gastric antral mucosa biopsies of patients spanning a cascade of gastric premalignant lesions and early gastric cancer (EGC) from the NCBI GEO database (GSE134520) [17] (supplementary Figs. S6B, C). Then, we obtained 34 clusters by utilizing principal component analysis (Fig. 7D). These biomarkers, i.e., FCGR3A, CD14, CTSB, LGMN, CD68, and CSF1R, were utilized to identify macrophage. Two clusters macrophage were obtained, i.e., Macrophage-01 (CTSB(+)CD68(+)) (cluster 25) and Macrophage-02 (CTSB(+)CD68(−)) (cluster 14) (Fig. 7D, E). GPR35 were found high expressed in Macrophage-01 (CTSB(+)CD68(+)) but very low expressed in Macrophage-02 (CTSB(+)CD68(−)) (Fig. 7E, F). These results suggested that GPR35 probably only impact functions for CTSB+ and CD68 + macrophage, such as infiltration level.

These sequencing cells were obtained from 13 biopsies, which spanned the cascade from gastritis to EGC [17]. Biopsies consisted of three wild superficial gastritis (NAG) biopsies, three chronic atrophic gastritis (CAG) biopsies, six intestinal metaplasia (IM) biopsies, and one EGC biopsy (Fig. 7G). The IM biopsies had three pylori-infected IM samples (IMW1, IMS1, and IMS2) and three uninfected IM samples (IMW2, IMS3, and IMS4). As shown in Fig. 7H, NAG and CAG biopsies has very low GPR35 level in CTSB+ and CD68 + macrophage. Meanwhile, a lot of cells for CTSB+ and CD68 + macrophage start expressing GPR35 when the stomach changes from NAG/CAG to IM stage, although the expression level of GPR35 is still very low. However, GPR35 in CTSB+ and CD68 + macrophage for EGC biopsy has the highest expression level among 13 biopsies (Fig. 7H). These findings determined that the GPR35 expression level in CTSB+ and CD68 + macrophage has a positive relation to the deterioration of the stomach, such as the stomach from NAG to EGC. Hence, GPR35 potentially acts as a biomarker for monitoring and early warning of primary gastric cancer.

## Discussion

Since overall high prevalence and high mortality rate for GC, it remains one of the leading cancers in the world and an important global healthcare problem [11, 18]. GC patients are largely threatened with unfavorable clinical prognosis owing to metastasis, drug resistance, and lack of biomarkers for early detection [2, 19]. Meanwhile, oGPCRs were known that they could act as potential biomarkers or pharmacological targets for many cancers [8]. In this study, an orphan GPCR, GPR35, were found closely affiliated with GC occurrence, development, and prognosis. Copy number variations and promoter methylation level fluctuations are not reasons for GPR35 high expression in GC tumors. In the GC tumors, GPR35 could be activated by enhancer release and retargeting event, which probably is a novel de-orphanization/activation method.

GPR35 is a Class A, rhodopsin-like G protein-coupled receptor which is predominantly expressed in immune and gastrointestinal tissue, with notable expression in the small intestine, colon, stomach, duodenum, and rectum [14, 15, 20]. For spontaneous and colitis-associated colon cancers, activation of the GPR35 pathway promotes tumor development mainly via two separate routes: one way to do is by directly augmenting proliferation in epithelial cells that express the receptor; other way to do is by coordinating macrophages’ ability to create a tumor-permissive environment [14, 15, 21]. However, GPR35 functions in GC tumors and the mechanisms by which GPR35 modulates the immune microenvironment remain unknown. Here, we found that a higher expression of GPR35 in tumors means a poorer prognosis of gastric cancer. Meanwhile, GPR35 promotes immune cell exhaustion (such as CD8 + T-cells and CD4 + memory T-cells), immune infiltration levels (such as T-cells and macrophage), and polarization of M2 macrophages in GC tumors. However, for immune infiltration levels, only GPR35 promoting macrophage infiltration level could lead to a bad prognostic for GC patients.

According to HR values of GPR35 in different clinical factors for gastric cancer, it further determined that ERR-activated GPR35 plays an important role in the progress of stomach adenocarcinoma development. Meanwhile, in the stomach-related diseases (NAG, CAG, IM, and EGC), the higher GPR35 expression level in CTSB+ and CD68 + macrophage, and the more deterioration of the stomach. Furthermore, GPR35 in CTSB+ and CD68 + macrophage for EGC had a notable high expression level when compared to those of other stomach diseases. These results suggested that GPR35 could be an early indicator of primary GC and a good druggable receptor for almost all clinical types of GC patients.

Taken together, high expression level for GPR35 in GC tissues could result in cancer cell proliferation and migration, and leads to some immune cells’ significance exhaustion, macrophage infiltration, and polarization of M2 macrophages. Then, these biological functions could lead to a poor prognosis of GC patients with high expression levels for GPR35. Furthermore, GPR35’s high expression in CTSB+ and CD68 + macrophage could be a genetic indicator for early warning of primary GC. Hence, our systematic study of ERR events in GPR35 and its biological functions in gastric cancer will serve as a valuable resource for exploring a novel activation pathway for oGPCRs and finding early genetic indicator and druggable receptor for GC patients.

## Materials and methods

### Identification of enhancers and promoters in gastric tissue

The protein-coding genes in the human genome were downloaded from the GENCODE [22]. Promoters were defined as regions located 3 kilo-base pairs (kb) upstream and 3 kb downstream of transcription start sites (TSSs) annotated in GENCODE [22].

Genomic regions of enhancers in the gastric tissue were derived from H3K27ac data generated by ENCODE project (ENCFF682YFS). Enhancers located in promoters and gene bodies of protein-coding genes were excluded.

### ChIP-seq data analysis

All of the ChIP-seq data were generated by the ENCODE Consortium [23]. The original reads were mapped to the human reference genome (GRCh37/hg19) using *Bowtie2* [24]. To identify ChIP-seq peak regions, we performed peak calling using MACS with the default parameters [25].

### TCGA data download, process, and analysis

The mRNA expression data and clinical data of STAD (GC) patients were downloaded from the TCGA database (https://genome-cancer.ucsc.edu/). These expression data were firstly normalized and differential expression analysis was then performed for SEMA3F by R package limma [26]. The *p* value <0.05 was considered statistically significant. According to clinical data, we estimated cumulative survival curves and overall survival rates using Kaplan–Meier curves. Then, the hazard ratio (HR) and corresponding 95% CIs were estimated based on Cox proportional hazard models. Indeed, higher HR values (HR >1.0) indicate a bad prognosis, while lower HR values (HR <1.0) indicate a good prognosis. *P* value was calculated by log-rank test. The mRNA expression data to explore the relationship between CAPN10 and GPR35 was downloaded from the European Genome–phenome Archive (EGA) database (accession number: EGAS00001002872) [27]. For the correlation analysis of GPR35 expression level and copy number data, the copy number data was extracted from the Broad TCGA Stomach Adenocarcinoma copy number dataset. Meanwhile, mutation and copy number variations of TCGA Stomach Adenocarcinoma were also compiled by using cBioPortal [28]. Transcript expression of GPR35 in the stomach and corresponding cancers were explored based on data from GTEx and TCGA database. Then, the mRNA alternative splicing situation for GPR35 was explored based on TSVdb (TCGA splicing variants database).

Furthermore, we downloaded a standardized pan-cancer dataset from the UCSC (https://xenabrowser.net/) database. Then, tumor mutational burden (TMB) was calculated by the TMB function in R package mafTools for pan-cancer. Meanwhile, purity values, EREG.EXPssn scores and RNAvalues scores for each type of cancer were referenced to the previous studies [29, 30]. Combined with expression of GPR35 in each cancer, the person correlation coefficient between GPR35 expression (log2(x + 0.001) transformation) and TMB/ purity / tumor stemness scores were computed, respectively.

### CRISPR/Cas9 system

The CRISPR/Cas9 system has been adapted as an efficient genome editing tool in laboratory animals such as mice, rats, and so on. The binding of CTCF to the CAPN promoter region was specifically knocked out by CRISPR based on JASPAR’s prediction of the binding of CTCF to the CAPN promoter region. The CTCF-binding domain for CAPN10 is chr2: 240587440-240588021 (hg38).

### Quantitative real-time PCR assay

Based on the manufacturer’s protocol, total RNAs were isolated using TRIzol (Invitrogen, CA, USA) and reversely transcribed into cDNAs (complementary DNAs) using a reverse transcription kit and the SYBR Green Master Mix kit (Takara, Otsu, Japan). The primer sequence for the GPR35 gene is 5′- CTCCCTGCGAGACACCTCAC-3′(F) and 5′-CTGATGCTCATGTACCTGT TGG -3′(R). Meanwhile, the primer sequence for the CAPN10 gene is 5′- GGTCTCAGAACCGAGTGAGGT-3′(F) and 5′- CCACGAAGTATGACTGT CACC -3′(R).

### Survival analysis for CAPN10 and GPR35 in GC

Kaplan–Meier plotter (http://kmplot.com/analysis/), an online database, could be utilized to access the effects of genes on survival in more than 20 cancer types, including GC [2]. Then, it was employed to conduct survival analysis for CAPN10 and GPR35 in GC. Log rank *p* value <0.05 was defined as statistically significant.

### DNA methylation analysis

TCGA DNA methylation data files for GPR53 in gastric cancer and matched normal tissues were collected from Genomic Data Commons. We utilized the Illumina Human Methylation 450k R annotation data package to map the Illumina methylation array probes to individual genes. We then retained those probes mapped to the corresponding promoter region. Median beta values were utilized when genes were with multiple probes. We then calculated the median beta value for GPR35 in each sample for calculating the overall methylation level in the promoter region. To examine the regulation of GPR35 expression by DNA methylation, we estimated the spearman correlation between DNA methylation beta values with mRNA expression for GPR35.

### Correlation analysis between genes expression in GC

Biomarkers for diverse immune cells in GC were downloaded from the CellMarker database (http://biocc.hrbmu.edu.cn/CellMarker/) [31]. Genes for RNA modifications, immunoregulatory, and immune checkpoints were downloaded from previous studies [29, 32]. Then, the expression associations between the genes of our interest were performed by spearman’s correlation analysis. The *p* < 0.05 represents statistically significant.

### Plasmids and siRNA transfection

The siRNA sequence for the GPR35 gene is 5′- AUGCGGCAGCA GAACACCCTT -3′. The plasmids and siRNA were transfected into cells with Lipofectamine 3000 based on the manufacturer’s instructions (Invitrogen, Grand Island, NY, USA). The siRNA was utilized to inhibit GPR35 expression.

### Cell viability assays

Firstly, three types of gastric cancer cells were utilized to investigate GPR35 expression levels. Then, one type gastric cancer cell with the highest GPR35 expression level among the three was selected to perform the following biological experiments, i.e., cell viability assays, Transwell migration, and cell-based wound healing assay. Then, for consideration diversity of gastric cancer cells, other gastric cancer cells was utilized to perform Flow cytometry and M2 macrophage polarization analysis.

Cell Counting Kit 8 (CCK8) provides a convenient and robust way of performing a cell viability assay for treated gastric cancer cells. The kit uses a water-soluble tetrazolium salt to quantify the number of live cells by producing an orange formazan dye upon bio-reduction in the presence of an electron carrier. The amount of formazan produced is directly proportional to the number of living cells and is measured by absorbance at 450 nm.

### Transwell migration assay

The migration assays were performed by utilizing the Transwell™ filter, a modified two-chamber plate with a pore size of 8 μm. The treated gastric cancer cells were seeded onto Transwell inserts (upper chamber). Meanwhile, the medium with 10% FBS configurations was added to the lower compartment. After 12/24 h of incubation at 37 °C, media within the Transwell inserts were carefully removed. Cells were fixed with 2% paraformaldehyde and then stained with crystal violet (Beyotime, Shanghai, China) for 30 min. Cells that did not migrate across the transwell membrane were then removed by gently wiping with a cotton swab. Migrated cells were imaged with an Olympus XC50 camera using the anaLYSIS software and processed using ImageJ software (National Institutes of Health, USA).

### Cell-based wound healing assay

Gastric cancer cells were first seeded into plates with 50–60% concentration. Then, these plates were treated with 5ul (1.2 ng) si-GPR35 or siNC for 24 h, after the cells have attached to the wall. Subsequently, we refreshed the medium and cultured for 24 h. Then, conventional pipette tip (10 µL tip) scratching was performed. Lastly, wound size measurements were performed using ImageJ software.

### Flow cytometry

Gastric cancer cell apoptosis after treatment was measured using a flow cytometer FACSCalibur (BD Biosciences). Cells (1 × 10^6^) were washed and then incubated with 5 μl of si-GPR35 or siNC. Next, the cells were washed and centrifuged, followed by analysis using a flow cytometer FACSCalibur (BD Biosciences).

### Immune cell infiltration level analysis

Infiltration levels for distinct immune cells in GC were quantified by using CIBERSORT and TIMER [33–35]. CIBERSORT is an analytical tool developed to provide an estimation of the abundances of member cell types in a mixed cell population based on gene expression data. Meanwhile, the TIMER is another tool for comprehensive analysis of tumor-infiltrating immune cells. Here, they were combined and utilized to analyze the correlation of GPR35 expression level with immune cell infiltration level in GC. *P* value <0.05 was considered statistically significant.

### M2 macrophage polarization

The THP-1 cell line was cultured with RPMI 1640 medium (Gibco, Thermo Fisher Scientific), containing 10% fetal bovine serum (Gibco, Thermo Fisher Scientific), 1% of penicillin G, and streptomycin sodium (Gibco, Thermo Fisher Scientific). All cell lines were purchased from Shanghai Institutes for Biological Sciences and incubated in 95% humidified air at the condition of 37 °C and 5% CO_2_. THP-1 cells were differentiated into M0 macrophages by incubation in 100 ng/ml PMA (Sigma-Aldrich) for 36 h.

Sg/nc-CTCF-bd-mkn45 and si/nc-GRP35-mkn45 cells were incubated at 37 °C for 2 days and centrifuged at 800 rpm for 5 min to collect the supernatant. The supernatant was mixed with freshly prepared 10% FBS at a ratio of 1:2 for the final conditioned medium.

M0 macrophages were treated with indicated conditioned medium for 48 h. For the surface marker analysis, cells were re-suspended in 0.1% BSA 1xPBS and stained with anti-human CD206 (FITC, Bio-Legend) at 4 °C for 20 min. Data were acquired by LSRFortessa (BD Bioscience) and analyzed with FACS Diva and FlowJo software.

### Analysis of scRNA-seq data

The single-cell RNA sequencing data were downloaded from the NCBI GEO database (GSE134520) [17]. The scRNA-seq data analysis was consistent with the previous study [17]. The Seurat function “FindVariableFeatures” was first utilized to identify the highly variable genes (HVGs). Then, the top 2000 HVGs were applied for data integration. The data were scaled using “ScaleData” and the first 40 principle components were chosen for auto-clustering analyses using “FindNeighbors” and “FindClusters” functions. For all cells, we identified clusters setting the resolution as 1.5. The clustering results were then visualized with the UMAP scatter plot. The marker genes of macrophage were downloaded from the CellMark database [31].

## Supplementary information

Supplementary materials

## Data Availability

The data supporting the conclusions of this article are presented within the article and its additional files.
