# Supplementary material for: ERR-activated GPR35 promotes immune infiltration level of macrophages in gastric cancer tissues
Source: Cell Death Discov. 2022 Nov 4;8:444. doi: 10.1038/s41420-022-01238-4 (PMC9636254; doi:10.1038/s41420-022-01238-4)
Supplement: Supplementary file 1 — Supplementary materials [file 41420_2022_1238_MOESM1_ESM.docx]

**ERR-activated GPR35 promotes immune infiltration level of macrophages in gastric cancer tissues**

Chuanjun Shu1,2#*, Can Wang^3^#, Saisai Cheng^3^#, XuanHuang^4^#, Jiahua Cui2, Wenchao Li^3^, Bin Xu^3^*

1Department of Bioinformatics, School of Biomedical Engineering and Informatics, Nanjing Medical University, Nanjing 211166, China.

2Department of Molecular Cell Biology & Toxicology, Center for Global Health, School of Public Health, Nanjing Medical University, Nanjing 211166, China.

3Department of Urology, Affiliated Zhongda Hospital of Southeast University, Nanjing, Jiangsu 210009, China.

4Reproductive Medical Center, Jinling Hospital Affiliated to Medical School of Nanjing University, Nanjing, Jiangsu 210002, China.

^#^These authors contribute equally to this study.

*Corresponding author: Email: Bin Xu: [njxbseu@seu.edu.cn](mailto:njxbseu@seu.edu.cn); Chuanjun Shu: [chuanjunshu@njmu.edu.cn](mailto:chuanjunshu@njmu.edu.cn).

**Running title:** ERR activation orphan GPCR GPR35

## Supplementary Figure S1 to S6:


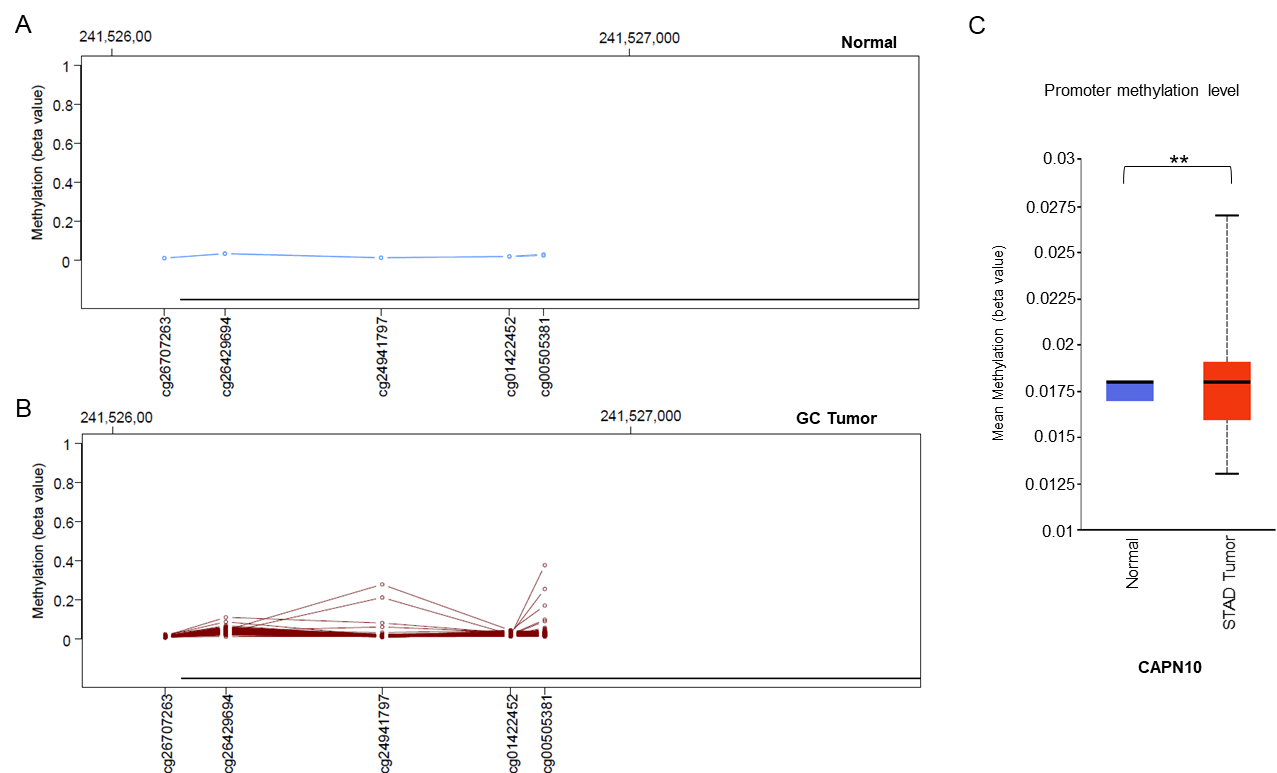


**Figure S1.** Promoter methylation level for CAPN10 in GC. (A) Promoter methylation levels (chr2: 241526000 − 241527500) of CAPN10 in normal (n=2) gastric tissues. (B) Promoter methylation levels (chr2: 241526000 − 241527500) of CAPN10 in GC tumors (n=339). (C) A significance different between promoter mean methylation in normal gastric tissues and STAD (GC) tumors.


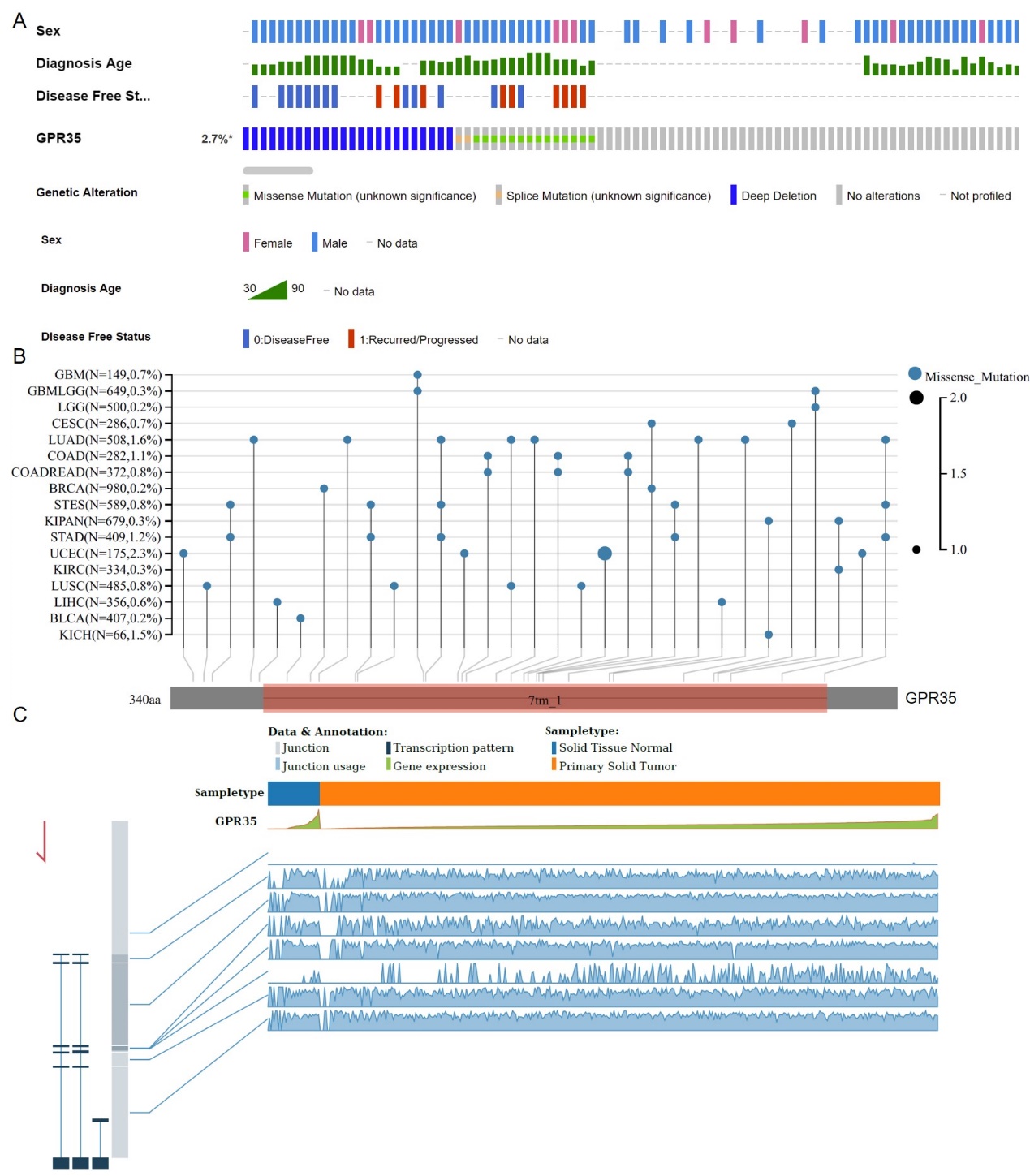


**Figure S2.** Landscape for GPR35 mutation and junction reads. (A) OncoPrint for GPR35 in GC tumors. (B) Landscape for GPR35 missense mutation in pan-cancer. (C) Landscape for junction usage for GPR35 in GC tissues and their corresponding normal solid tissues.


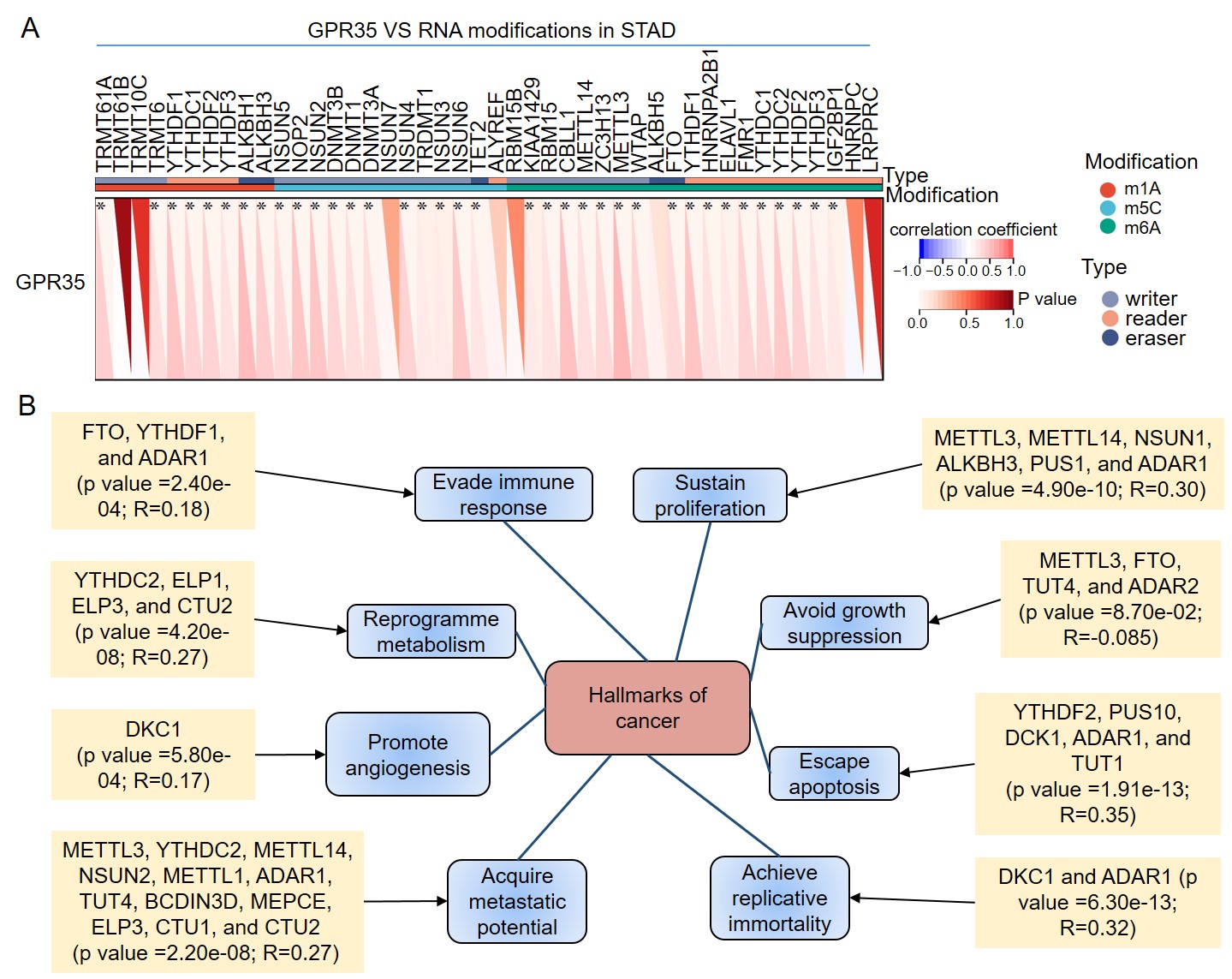


**Figure S3.** The correlation coefficient between GPR35 and signature genes for RNA modifications. (A) Expression correlation between RNA modification genes and GPR35 in STAD tissues. (B) In STAD, GPR35 may be play a role during RNA modification genes influence hallmarks of cancer.


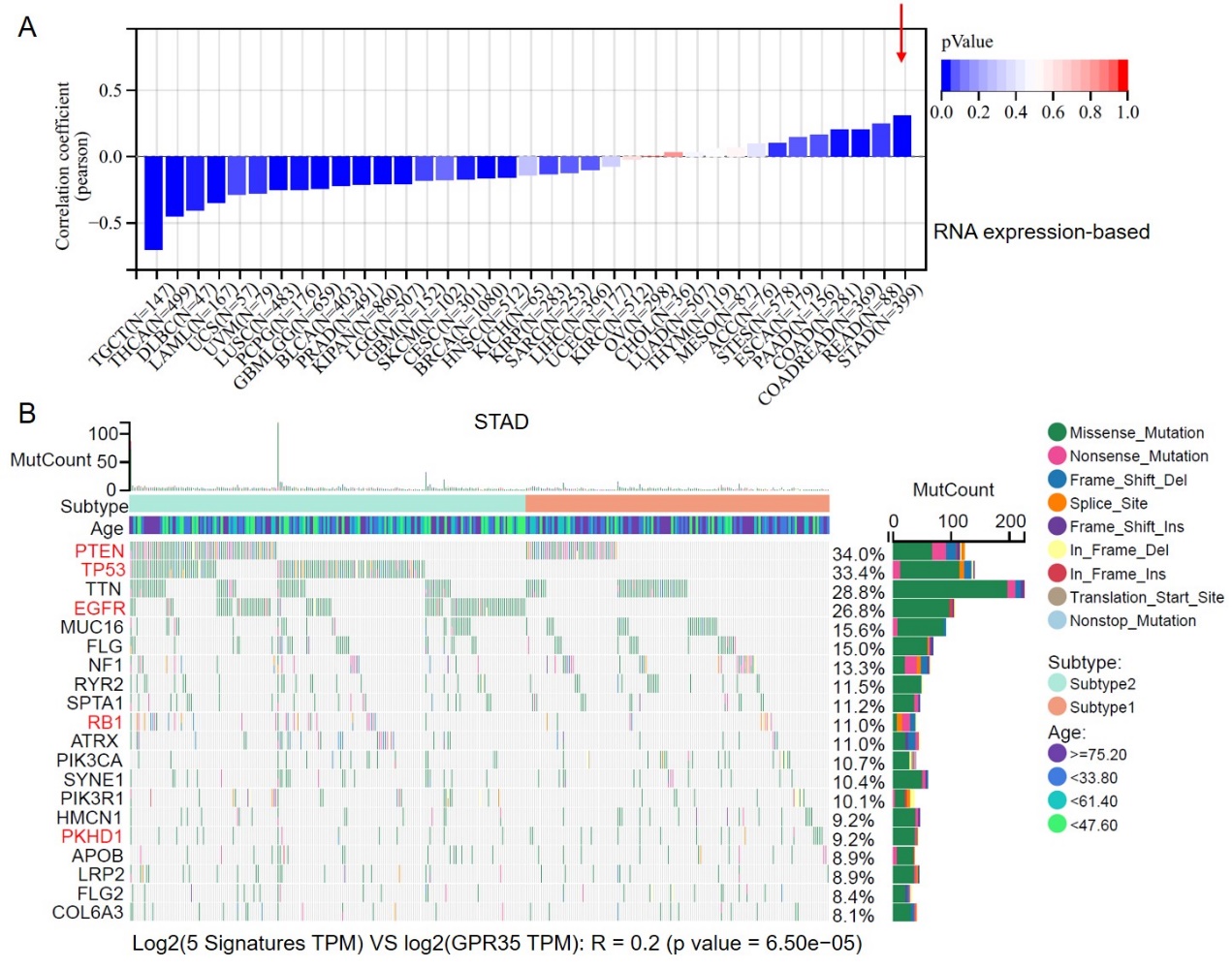


**Figure S4.** GPR35 expression correlation coefficient with tumor stemness and Mutation genes in GC. (A) Landscape for GPR35 correlation coefficient with stemness of cancer cells in pan-cancer. The tumor stemness was presented by utilizing RNAss. (B) The relationship for expression between GPR35 and genes with high mutation frequency in STAD.


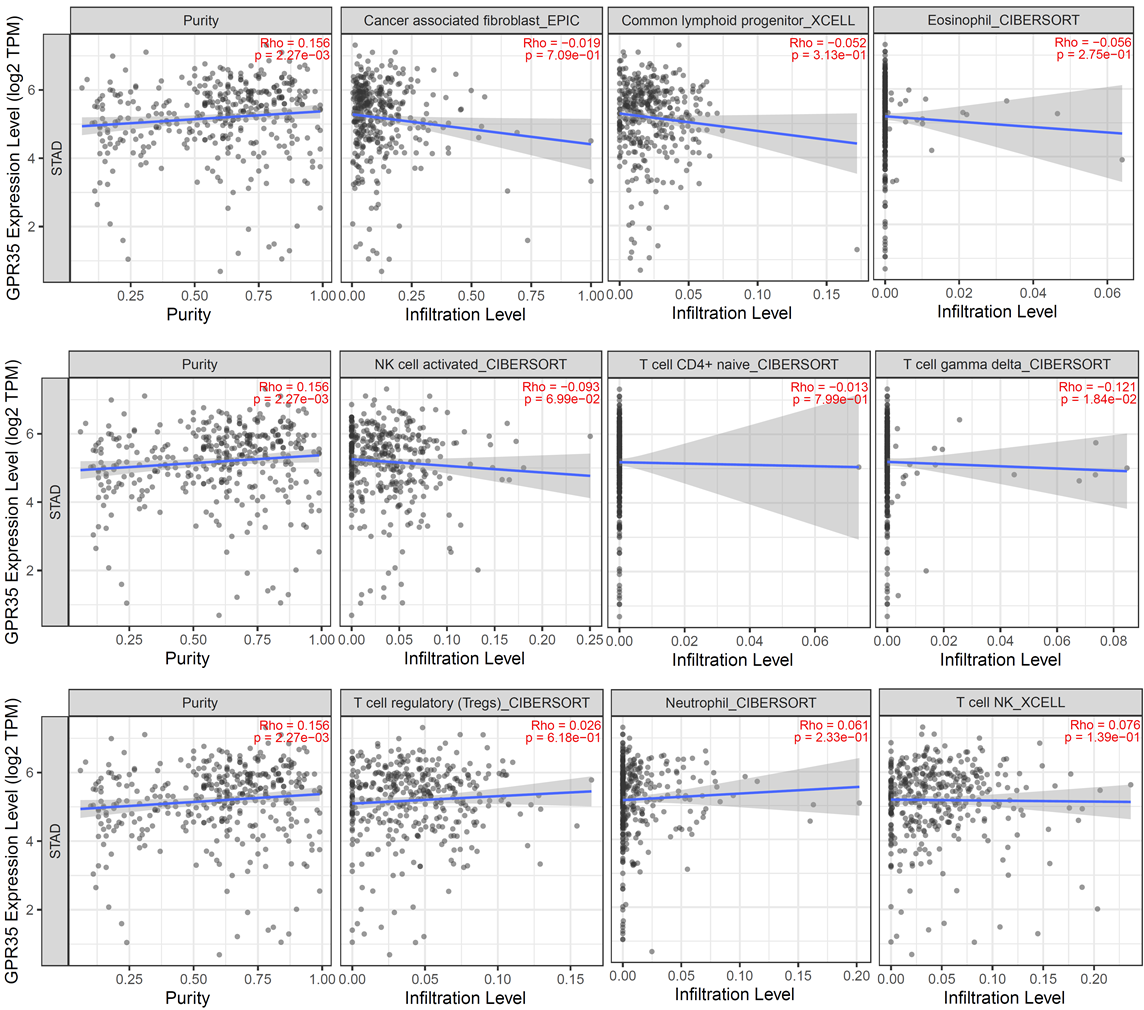


**Figure S5.** GPR35 no significance links infiltration of partly immune cells in GC tumors. The associations between GPR35 expression level and immune cells infiltration level were performed by spearman’s correlation analysis. P value <0.05 was considered as statistically significant.


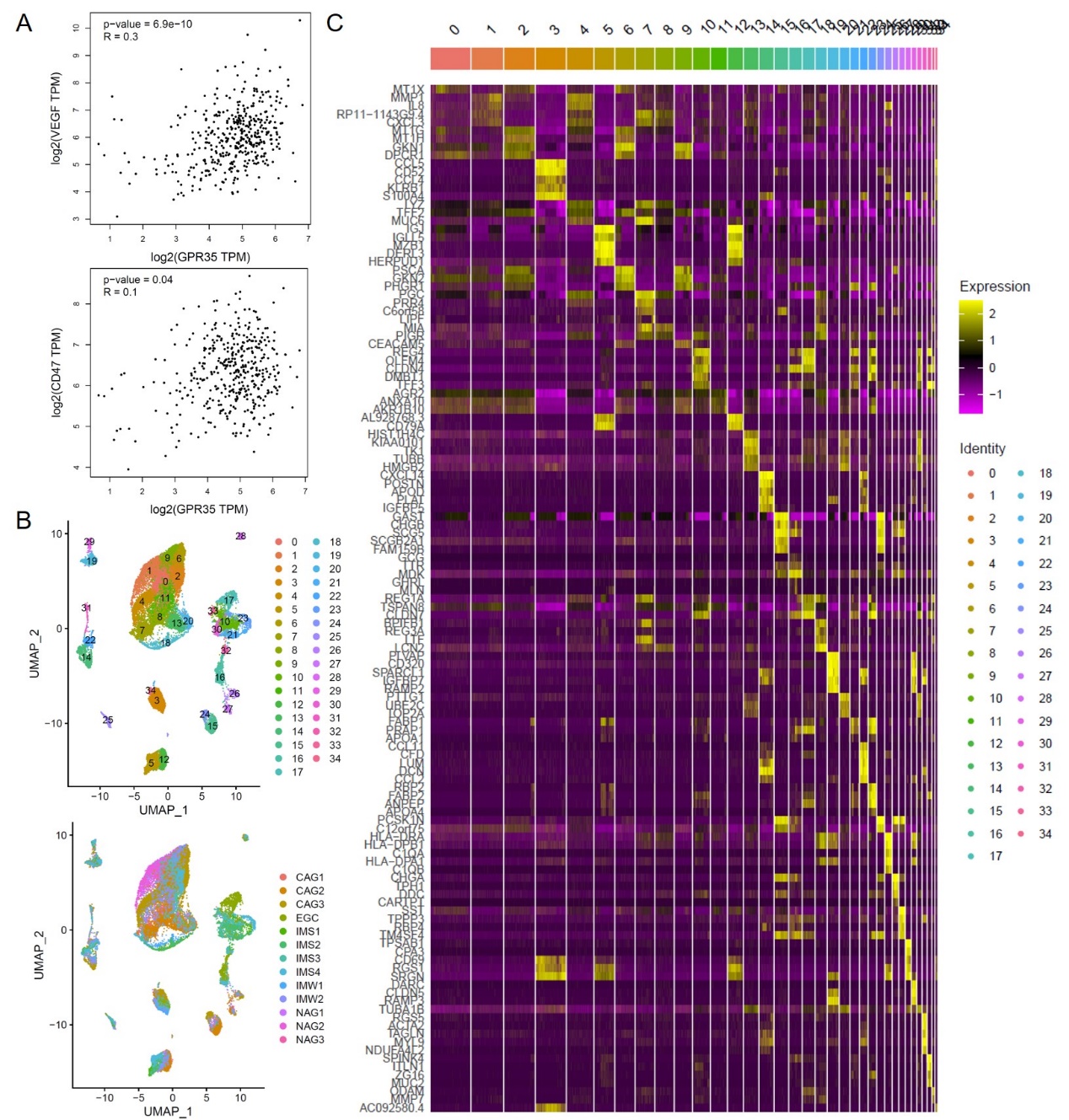


**Figure S6. Top five genes in different clusters.** (A) Correlation between GPR35 and tumor-associated macrophage markers. (B) UMAP plot for 35 clusters and 13 samples. (C) Top 5 genes in different clusters.

## Supplementary Table S1 to S3:

**Table S1.** ERR events in stomach

| **Gene-CP** | **Gene-AP** | **enhancer (hg38)** |
| --- | --- | --- |
| MXD3 | PRELID1 | chr5_177339050_177339888 |
| MICB | XXbac-BPG181B23 | chr6_31562996_31563276 |
| HCG4P5 | ZNRD1ASP | chr6_29963963_29968759 |
| AP000350 | GSTT2 | chr22_23919167_23920601 |
| LINC01547 | AP001505 | chr21_44986330_44987907 |
| NUDT18 | FAM160B2 | chr8_22117695_22117930 |
| RP11-550I24 | KRT18P34 | chr3_157110128_157111256 |
| CLCNKA | CLCNKB | chr1_16011966_16012271 |
| FIS1 | CLDN15 | chr7_101243001_101245741 |
| HCG27 | HLA-C | chr6_31246281_31246849 |
| GBAP1 | RUSC1-AS1 | chr1_155203552_155203787 |
| TSSK6 | YJEFN3 | chr19_19457729_19465678 |
| CALML6 | RP1-140A9 | chr1_1906783_1907330 |
| ZSCAN12P1 | ZSCAN26 | chr6_28104279_28105548 |
| MICB | MICA | chr6_31562996_31563276 |
| ERV3-1 | INTS4P1 | chr7_65046639_65046955 |
| HLA-DQB1 | HLA-DQB2 | chr6_32622554_32623136 |
| ABHD12 | ENTPD6 | chr20_25540202_25540884 |
| HMGN4 | ZNF322 | chr6_26553112_26555395 |
| RP11-44F14 | AKTIP | chr16_53510823_53511459 |
| RP11-344N10 | DNAJC9 | chr10_73118360_73118611 |
| ATP23 | EEF1AKMT3 | chr12_57886416_57886971 |
| PDZD8 | RP11-129M16 | chr10_117397798_117398097 |
| TAS2R5 | TAS2R4 | chr7_141694643_141696871 |
| RP11-158I9 | TREH | chr11_118875843_118876986 |
| ATOH7 | PBLD | chr10_68315331_68315746 |
| GSDMB | GSDMA | chr17_39772541_39774562 |
| BTBD6 | BRF1 | chr14_105293951_105294558 |
| REELD1 | RP11-6L6 | chr4_146235052_146236939 |
| ZNF589 | NME6 | chr3_48400955_48404954 |
| ZNF273 | CCT6P3 | chr7_65001624_65002035 |
| HCG4P5 | HLA-W | chr6_29963963_29968759 |
| ZNF79 | RPL12 | chr9_127526553_127529676 |
| MFAP2 | CROCC | chr1_17067237_17067873 |
| HLA-W | HCG4P5 | chr6_30122290_30123759 |
| HSD17B1 | NAGLU | chr17_42670251_42670862 |
| ZNF7 | RPL8 | chr8_144848802_144849750 |
| RP11-395L14 | SLC35F5 | chr2_113785377_113785909 |
| UHRF1BP1 | SNRPC | chr6_34961421_34961865 |
| RP4-756H11 | GS1-124K5 | chr7_66813540_66813775 |
| MRPL45P2 | TBKBP1 | chr17_47667211_47667543 |
| APIP | PDHX | chr11_35047104_35048423 |
| HLA-DRB1 | HLA-DQB2 | chr6_32622554_32623136 |
| PI4KA | SERPIND1 | chr22_20951881_20952209 |
| TTC38 | PKDREJ | chr22_46442532_46445237 |
| TMEM220 | CTC-297N7 | chr17_10785828_10786606 |
| AGRN | RP11-465B22 | chr1_1032475_1033075 |
| MICA | MIR6891 | chr6_31562996_31563276 |
| AC024560 | RP11-496H1 | chr3_197718576_197719206 |
| MIF | GSTT2 | chr22_23919167_23920601 |
| TRIM37 | SKA2 | chr17_59002228_59002660 |
| MAGOH2P | CTC-297N7 | chr17_10785828_10786606 |
| NPIPB2 | GSPT1 | chr16_12057839_12058186 |
| MIR6891 | MICA | chr6_31411633_31411895 |
| HLA-DQB1-AS1 | HLA-DQB2 | chr6_32622554_32623136 |
| RP11-54O7 | RP11-465B22 | chr1_1156242_1161390 |
| SLC9A8 | RNF114 | chr20_49951676_49951968 |
| CCT6P3 | INTS4P1 | chr7_65001624_65002035 |
| MIR6891 | XXbac-BPG181B23 | chr6_31411633_31411895 |
| PPP1CB | TRMT61B | chr2_28951371_28953524 |
| DCAKD | ACBD4 | chr17_45201342_45201831 |
| ORMDL1 | OSGEPL1-AS1 | chr2_189951460_189951792 |
| MAPK3 | YPEL3 | chr16_30166478_30166716 |
| RP11-496H1 | AC024560 | chr3_197718576_197719206 |
| POLR1B | RGPD8 | chr2_112630203_112632330 |
| GSTA2 | GSTA1 | chr6_52787406_52787657 |
| HCG27 | XXbac-BPG181B23 | chr6_31246281_31246849 |
| FAM228B | FAM228A | chr2_23951491_23951768 |
| HLA-DQB1 | HLA-DOB | chr6_32622554_32623136 |
| GMPPB | ACTL11P | chr3_49665318_49667004 |
| HLA-DQA1 | HLA-DQB2 | chr6_32622554_32623136 |
| DDX11L2 | SLC35F5 | chr2_113785377_113785909 |
| PCDHB11 | PCDHB16 | chr5_141084655_141085117 |
| PRPSAP2 | TVP23B | chr17_18866879_18867225 |
| STAG3L4 | RP11-166O4 | chr7_67143645_67143895 |
| FANCA | LINC02166 | chr16_89826959_89830102 |
| SLC20A1 | AC012442 | chr2_112641093_112641647 |
| SPATA5L1 | CTD-2651B20 | chr15_45454069_45458890 |
| PMS1 | OSGEPL1-AS1 | chr2_189641205_189641812 |
| KRT18P34 | RP11-550I24 | chr3_157247918_157248679 |
| CYP2D7 | CYP2D6 | chr22_42218119_42218456 |
| C22orf34 | RP1-29C18 | chr22_49670182_49670500 |
| RRP7BP | RRP7A | chr22_42622941_42625214 |
| RP11-797H7 | GTF2IP14 | chr7_65001624_65002035 |
| HCG9 | RNF39 | chr6_30097349_30098633 |
| HLA-C | MICA | chr6_31246281_31246849 |
| HLA-DRB1 | HLA-DQA2 | chr6_32622554_32623136 |
| XXbac-BPG181B23 | HCG27 | chr6_31562996_31563276 |
| YJEFN3 | TSSK6 | chr19_19457729_19465678 |
| LSMEM1 | IFRD1 | chr7_112462079_112463182 |
| HCP5B | HLA-W | chr6_29963963_29968759 |
| CNTNAP1 | HSD17B1 | chr17_42670251_42670862 |
| TCTN2 | DDX55 | chr12_123695681_123697204 |
| PSCA | LYNX1 | chr8_142842220_142842527 |
| HLA-W | HLA-K | chr6_30122290_30123759 |
| PRH1 | TAS2R64P | chr12_11071427_11071793 |
| SELENOO | MAPK12 | chr22_50219658_50220237 |
| SAAL1 | SERGEF | chr11_17922224_17922477 |
| NUP133 | HMGN2P19 | chr1_229582037_229582706 |
| ZNF33B | LINC00839 | chr10_42644963_42645873 |
| KLHL7-DT | GPNMB | chr7_23246127_23248572 |
| HLA-W | HLA-J | chr6_30122290_30123759 |
| HCP5B | POLR1H | chr6_29963963_29968759 |
| RP4-584D14 | ZBED6CL | chr7_150354686_150356548 |
| NDUFV3 | CBS | chr21_42991984_42993102 |
| METTL27 | ABHD11-AS1 | chr7_73905211_73905711 |
| WFDC10B | DNTTIP1 | chr20_45867510_45867773 |
| VXN | ADHFE1 | chr8_66506983_66508609 |
| FAM149B1 | CFAP70 | chr10_73240253_73240503 |
| GS1-124K5 | RP4-756H11 | chr7_66636120_66636366 |
| ZFP57 | HCG4 | chr6_29648998_29650250 |
| RP11-554A11 | MRGPRF-AS1 | chr11_69034632_69036450 |
| SF3B3 | IL34 | chr16_70589707_70592534 |
| HLA-V | HLA-K | chr6_29963963_29968759 |
| FBXL13 | LRRC17 | chr7_103129638_103130778 |
| CPNE1 | RPL36P4 | chr20_35796783_35797413 |
| HLA-DQA1 | HLA-DQA2 | chr6_32622554_32623136 |
| SLC35E4 | PES1 | chr22_30650388_30651228 |
| CRAT | RP11-247A12 | chr9_129197638_129199511 |
| DNTTIP1 | WFDC10B | chr20_45867510_45867773 |
| SMUG1 | FLJ12825 | chr12_54140438_54143385 |
| AMIGO1 | SYPL2 | chr1_109532174_109533088 |
| CYP2D6 | CYP2D7 | chr22_42218119_42218456 |
| BTNL8 | BTNL3 | chr5_180889392_180889925 |
| IFI27L2 | IFI27L1 | chr14_94113005_94115651 |
| BNIP3P11 | SEPHS1P1 | chr7_64558272_64558689 |
| SFT2D1 | HNRNPA1P49 | chr6_166330549_166330827 |
| ZNF641 | RP11-370I10 | chr12_48296461_48297158 |
| CTD-2336O2 | CTD-2336O2 | chr8_1785778_1786505 |
| RP11-632K20 | RP11-632K20 | chr15_32659194_32660591 |
| IL18 | BCO2 | chr11_112160082_112160490 |
| AP001469 | YBEY | chr21_46393719_46394291 |
| BTN3A2 | BTN3A1 | chr6_26553112_26555395 |
| ZNF117 | INTS4P1 | chr7_65046639_65046955 |
| HLA-C | HCG27 | chr6_31246281_31246849 |
| SLC25A27 | TDRD6 | chr6_46507257_46510701 |
| GGA2 | DCTN5 | chr16_23508644_23510983 |
| ZBED6CL | RP4-584D14 | chr7_150354686_150356548 |
| ZNF322 | HMGN4 | chr6_26553112_26555395 |
| TMED5 | DR1 | chr1_93322607_93326189 |
| AC093162 | CAPG | chr2_85412699_85415195 |
| HLA-DQB1-AS1 | HLA-DOB | chr6_32622554_32623136 |
| MASTL | ANKRD26 | chr10_27252391_27253113 |
| SULT1A2 | NPIPB9 | chr16_28609119_28610955 |
| NUP85 | MRPS7 | chr17_75400914_75401149 |
| MPI | ULK3 | chr15_75045657_75048774 |
| LINC00839 | ZNF33B | chr10_42644963_42645873 |
| RP11-989F5 | STX2 | chr12_130826984_130828099 |
| NELFB | STPG3 | chr9_137281674_137281932 |
| SMIM24 | C19orf71 | chr19_3471149_3471403 |
| AP000351 | DDT | chr22_23919167_23920601 |
| NQO2 | RP1-40E16 | chr6_3019288_3019765 |
| PLA2G6 | BAIAP2L2 | chr22_38186262_38186661 |
| AGER | NOTCH4 | chr6_32244418_32245347 |
| KCTD21-AS1 | NDUFC2 | chr11_78168171_78170333 |
| RP11-46C24 | SLC22A31 | chr16_89246991_89247911 |
| ZNF417 | ZNF814 | chr19_57800918_57806967 |
| REEP5 | SRP19 | chr5_112963424_112964578 |
| SRA1 | WDR55 | chr5_140628458_140628847 |
| NDUFC2 | KCTD21-AS1 | chr11_78168171_78170333 |
| RP11-90P13 | AC093627 | chr7_141435_144676 |
| EEF1DP4 | CCT6P3 | chr7_65001624_65002035 |
| RP11-212P7 | RP11-274B21 | chr7_128560920_128561369 |
| RP11-334C17 | SGSH | chr17_80253569_80256440 |
| HCG9 | HCG4P3 | chr6_30097349_30098633 |
| TULP2 | HSD17B14 | chr19_48907556_48908688 |
| SNRPC | UHRF1BP1 | chr6_34816473_34817197 |
| RP11-493E3 | AP3S2 | chr15_89865372_89866316 |
| ZNF786 | RP4-800G7 | chr7_149209101_149209480 |
| MTHFSD | RP11-463O9 | chr16_86530386_86531786 |
| RBM23 | HAUS4 | chr14_22951235_22951945 |
| IFT122 | RPL32P3 | chr3_129557507_129562277 |
| POMZP3 | UPK3B | chr7_76583494_76584362 |
| RP11-493E12 | AHSA2P | chr2_61355913_61356772 |
| GMPPB | UBA7 | chr3_49665318_49667004 |
| RPL10 | FAM3A | chrX_154383823_154384184 |
| HLA-C | HLA-S | chr6_31246281_31246849 |
| RP5-1159O4 | MIOS-DT | chr7_7562332_7562782 |
| RPS26 | SUOX | chr12_56084622_56088167 |
| NMRAL1 | CDIP1 | chr16_4528825_4530073 |
| POU5F1 | HLA-C | chr6_31246281_31246849 |
| AC000078 | TXNRD2 | chr22_19881458_19882371 |
| RHCE | SDHDP6 | chr1_25502912_25503825 |
| RP11-325L12 | SPG21 | chr15_65069506_65072850 |
| SGTA | ZNF554 | chr19_2774648_2776594 |
| RP11-110I1 | HMBS | chr11_119080685_119081209 |
| HIBCH | INPP1 | chr2_190499567_190501276 |
| THEM4 | S100A10 | chr1_151981814_151982438 |
| MRPS7 | NUP85 | chr17_75458381_75460835 |
| RP11-6L6 | REELD1 | chr4_146192329_146193015 |
| LIPT1 | LYG1 | chr2_99324509_99325151 |
| HLA-K | HLA-J | chr6_30122290_30123759 |
| RP11-157J24 | RP11-157J24 | chr6_1554319_1555449 |
| RP1-29C18 | C22orf34 | chr22_49670182_49670500 |
| RP11-109L13 | AP000892 | chr11_117225827_117226967 |
| TRAPPC12-AS1 | TRAPPC12 | chr2_3488930_3489165 |
| SURF6 | SURF1 | chr9_133422103_133423890 |
| CLCNKB | CLCNKA | chr1_16171447_16185386 |
| NUTM2A | NUTM2D | chr10_87090151_87090971 |
| NBPF3 | NBPF2P | chr1_21546436_21547231 |
| XRRA1 | RP11-147I3 | chr11_75143457_75143961 |
| COQ6 | ENTPD5 | chr14_74064777_74065327 |
| LY6K | LYNX1 | chr8_142732712_142732947 |
| DDX55 | TCTN2 | chr12_123695681_123697204 |
| MIR6891 | HLA-C | chr6_31411633_31411895 |
| AF001548 | NDE1 | chr16_15775822_15777674 |
| RP11-344N10 | DNAJC9-AS1 | chr10_73118360_73118611 |
| CCDC154 | CLCN7 | chr16_1378522_1380061 |
| CTD-3025N20 | ARMC1 | chr8_65760770_65762637 |
| ALOX15P1 | KIAA0753 | chr17_6592539_6592933 |
| WEE2-AS1 | TAS2R5 | chr7_141694643_141696871 |
| HSD17B14 | BCAT2 | chr19_48907556_48908688 |
| RNASEH2B | RNASEH2B-AS1 | chr13_50916405_50917972 |
| HLA-A | HLA-J | chr6_30097349_30098633 |
| HLA-F | IFITM4P | chr6_29648998_29650250 |
| HAUS4 | RP11-298I3 | chr14_22951235_22951945 |
| PSORS1C3 | HLA-C | chr6_31246281_31246849 |
| YBEY | PCNT | chr21_46425066_46425938 |
| DGCR2 | DGCR5 | chr22_19108756_19111028 |
| ZNF708 | CTD-2561J22 | chr19_21504710_21506711 |
| OCEL1 | MRPL34 | chr19_17234898_17237513 |
| LIAS | RPL9 | chr4_39501396_39501856 |
| CDC25A | NME6 | chr3_48267776_48269306 |
| ZNF738 | LINC00664 | chr19_21321954_21322700 |
| ZNF814 | ZNF417 | chr19_57800918_57806967 |
| RPL12 | ZNF79 | chr9_127548252_127549201 |
| HLA-DOB | TAP2 | chr6_32981684_32983945 |
| TREH | RP11-158I9 | chr11_118875843_118876986 |
| GTF2IP14 | INTS4P1 | chr7_65251945_65252713 |
| CCHCR1 | HCG27 | chr6_31246281_31246849 |
| RPS23 | ATP6AP1L | chr5_82356788_82357339 |
| YBEY | AP001469 | chr21_46425066_46425938 |
| CTD-2651B20 | SPATA5L1 | chr15_45454069_45458890 |
| CTD-3116E22 | ZNF846 | chr19_9767577_9769577 |
| DCAKD | NMT1 | chr17_45201342_45201831 |
| UBE2I | CCDC154 | chr16_1302850_1304218 |
| RP1-40E16 | RIPK1 | chr6_2940546_2940885 |
| RIBC2 | FAM118A | chr22_45440844_45441436 |
| EFCAB2 | RP11-156E8 | chr1_245091491_245092048 |
| STPG3-AS1 | NELFB | chr9_137267757_137270336 |
| CCHCR1 | POU5F1 | chr6_31246281_31246849 |
| XXbac-BPG283O16 | HLA-L | chr6_30322407_30322753 |
| SULT1C3 | LINC01594 | chr2_108295855_108296461 |
| RP11-350J20 | LRP11 | chr6_149858912_149859295 |
| RIPK3 | DHRS1 | chr14_24178484_24181142 |
| DDX42 | FTSJ3 | chr17_63834469_63835958 |
| SERGEF | SAAL1 | chr11_17922224_17922477 |
| ABCF1 | MDC1 | chr6_30611240_30611578 |
| GSTA1 | GSTA2 | chr6_52787406_52787657 |
| SIVA1 | AKT1 | chr14_104746169_104748994 |
| THBS3 | GBAP1 | chr1_155203552_155203787 |
| NME1 | NME2 | chr17_51298010_51299142 |
| LA16c-325D7 | PRSS30P | chr16_2905820_2906892 |
| CTD-2651B20 | CTD-2651B20 | chr15_45162314_45163612 |
| AC011380 | SRA1 | chr5_140612431_140613002 |
| TIPIN | DIS3L | chr15_66472069_66472769 |
| RP11-298I3 | HAUS4 | chr14_22951235_22951945 |
| ZNF34 | RPL8 | chr8_144877896_144879124 |
| TAS2R4 | TAS2R5 | chr7_141694643_141696871 |
| SULT1C2 | LINC01594 | chr2_108297804_108298259 |
| SHMT1 | FAM106A | chr17_18356288_18357272 |
| RP11-43F13 | CTD-2012J19 | chr5_1567514_1568430 |
| DEPTOR | DSCC1 | chr8_120016916_120018829 |
| LINC02640 | PBLD | chr10_68315331_68315746 |
| TMC4 | NDUFA3 | chr19_54164319_54164864 |
| FTSJ3 | DDX42 | chr17_63834469_63835958 |
| ZDHHC20P1 | MICE | chr6_29648998_29650250 |
| MMP24OS | GDF5 | chr20_35269095_35269756 |
| SERPIND1 | PI4KA | chr22_20938434_20938869 |
| RP11-342K6 | MRPL19 | chr2_75560738_75561335 |
| HEXD | RP13-20L14 | chr17_82495603_82498594 |
| UVSSA | CTBP1-DT | chr4_1351647_1352074 |
| HMBS | VPS11 | chr11_119080685_119081209 |
| ARIH2 | QRICH1 | chr3_48939945_48940701 |
| TATDN3 | FLVCR1-DT | chr1_212832320_212835061 |
| LTB4R | ADCY4 | chr14_24328852_24329824 |
| ZFP57 | RPL23AP1 | chr6_29648998_29650250 |
| AHSA2P | RP11-493E12 | chr2_61327376_61327664 |
| PGAM1P8 | SLC22A20P | chr11_65277502_65277766 |
| KNSTRN | IVD | chr15_40421669_40422184 |
| SEPHS1P1 | CCT6P3 | chr7_65001624_65002035 |
| RPL36P4 | CEP250 | chr20_35738054_35738768 |
| BIVM | METTL21EP | chr13_102892831_102893349 |
| A2MP1 | OVOS | chr12_9244782_9245064 |
| CCDC32 | RP11-64K12 | chr15_40744056_40744412 |
| TMED9 | B4GALT7 | chr5_177677654_177678000 |
| DNLZ | SNAPC4 | chr9_136478507_136479333 |
| RP11-365P13 | ANKRD10-IT1 | chr13_110910388_110910820 |
| EXOC3 | SLC9A3-AS1 | chr5_458602_458972 |
| AP000350 | DDT | chr22_23919167_23920601 |
| ZFP57 | HLA-V | chr6_29648998_29650250 |
| GBAP1 | THBS3 | chr1_155203552_155203787 |
| AC007278 | IL18R1 | chr2_102427206_102427441 |
| IL17RA | CECR7 | chr22_17170947_17172530 |
| HLA-W | HCG4B | chr6_30122290_30123759 |
| FADS1 | FADS2 | chr11_61841988_61842348 |
| HCG9 | ZNRD1ASP | chr6_30097349_30098633 |
| NUDT13 | FAM149B1 | chr10_73240253_73240503 |
| GSPT1 | NPIPB2 | chr16_11912406_11912774 |
| GNB3 | LRRC23 | chr12_6880031_6881243 |
| RP11-129M16 | PDZD8 | chr10_117371193_117371485 |
| ERMARD | WDR27 | chr6_169697660_169697920 |
| RGS11 | MRPL28 | chr16_325706_328986 |
| ABHD11-AS1 | METTL27 | chr7_73886151_73887491 |
| RP5-908M14 | RP5-908M14 | chr20_62380408_62382972 |
| ALG11 | NEK3 | chr13_51999168_52000097 |
| EXOC2 | RP11-532F6 | chr6_699792_700054 |
| VAMP1 | TAPBPL | chr12_6547038_6548090 |
| HMGB1P31 | ACYP2 | chr2_54114581_54116390 |
| MICE | HLA-U | chr6_29648998_29650250 |
| U3 | PPID | chr4_158808627_158812781 |
| FAM228A | FAM228B | chr2_24364414_24366296 |
| SLC22A20P | AP003068 | chr11_65277502_65277766 |
| IL34 | SF3B3 | chr16_70646560_70647040 |
| SLC7A9 | NUDT19-DT | chr19_32988243_32988941 |
| RP11-182J1 | NMB | chr15_84650103_84650695 |
| AP3S2 | RP11-493E3 | chr15_89865372_89866316 |
| XXbac-BPG283O16 | HCG18 | chr6_30322407_30322753 |
| RPSA | CX3CR1 | chr3_39414219_39415313 |
| CTB-50L17 | UBXN6 | chr19_4396555_4396994 |
| TYW1 | LINC02604 | chr7_67143645_67143895 |
| IL18R1 | AC007278 | chr2_102427206_102427441 |
| SLC28A2 | CTD-2651B20 | chr15_45248290_45249184 |
| MASTL | ACBD5 | chr10_27252391_27253113 |
| FADS2 | FADS1 | chr11_61841988_61842348 |
| SP2-DT | PNPO | chr17_47983447_47984354 |
| RP1-179N16 | PNPLA1 | chr6_36246644_36246972 |
| HLA-U | HLA-J | chr6_30122290_30123759 |
| HLA-K | ZNRD1ASP | chr6_30122290_30123759 |
| PTPN21 | RP11-507K2 | chr14_88667636_88668235 |
| AGAP4 | AGAP10P | chr10_46024887_46025642 |
| POLR1A | MRPL35 | chr2_86302165_86302400 |
| RP3-425C14 | HSF2 | chr6_122609464_122611534 |
| ZNF846 | CTD-3116E22 | chr19_9767577_9769577 |
| THEM6 | LYNX1 | chr8_142756634_142757640 |
| CFDP1 | RP11-252K23 | chr16_75389682_75390418 |
| CTD-2260A17 | ERAP1 | chr5_96978580_96978823 |
| VPS9D1 | CDK10 | chr16_89826959_89830102 |
| ACOT2 | ACOT1 | chr14_73526296_73526746 |
| RP11-219D15 | ACTG1P1 | chr3_139532929_139533417 |
| PPP1R14B | PRDX5 | chr11_64334702_64335807 |
| ARMC10 | NAPEPLD | chr7_103129638_103130778 |
| RP11-465B22 | RP11-54O7 | chr1_1156242_1161390 |
| RPL36P4 | CPNE1 | chr20_35738054_35738768 |
| LINC01594 | SULT1C2 | chr2_108297804_108298259 |
| STX2 | RP11-989F5 | chr12_130826984_130828099 |
| AC012442 | SLC20A1 | chr2_112707636_112708304 |
| TOP1MT | RHPN1 | chr8_143334108_143335372 |
| EFTUD2 | DBF4B | chr17_44871896_44872670 |
| PKDREJ | TTC38 | chr22_46390707_46391550 |
| POU5F1 | PSORS1C2 | chr6_31246281_31246849 |
| PSORS1C1 | HLA-C | chr6_31246281_31246849 |
| SRP19 | REEP5 | chr5_112963424_112964578 |
| PSORS1C1 | XXbac-BPG299F13 | chr6_31246281_31246849 |
| INPP1 | HIBCH | chr2_190466741_190470676 |
| CBR3 | CBR3-AS1 | chr21_36141279_36141514 |
| GAREM2 | ADGRF3 | chr2_26273324_26275692 |
| DGCR11 | DGCR5 | chr22_19108756_19111028 |
| FARSA | CALR | chr19_12984318_12986372 |
| RPL8 | ZNF34 | chr8_144877896_144879124 |
| CDK11B | RP1-283E3 | chr1_1715748_1716074 |
| RP11-259G18 | NSFP1 | chr17_46286374_46286676 |
| COA8 | XRCC3 | chr14_103733190_103737047 |
| DDX55 | ATP6V0A2 | chr12_123695681_123697204 |
| CDK10 | TCF25 | chr16_89853172_89853407 |
| HLA-DQB1 | HLA-DQA2 | chr6_32622554_32623136 |
| MUC20 | MUC20P1 | chr3_195892189_195897762 |
| IL11RA | RP11-195F19 | chr9_34742344_34742678 |
| TVP23B | PRPSAP2 | chr17_18919369_18920626 |
| UPK3B | POMZP3 | chr7_76611518_76611753 |
| NDUFA3 | TMC4 | chr19_54164319_54164864 |
| C1QTNF9B | MIPEP | chr13_23725723_23726186 |
| DPH7 | PNPLA7 | chr9_137673751_137674618 |
| TAF1A-AS1 | RP11-378J18 | chr1_222598976_222602176 |
| TDRD6 | SLC25A27 | chr6_46520575_46521558 |
| HAAO | FTOP1 | chr2_42785573_42787064 |
| UBE2D3-AS1 | LRRC37A15P | chr4_103011296_103013239 |
| SRA1 | AC011380 | chr5_140628458_140628847 |
| AC093627 | RP11-90P13 | chr7_141435_144676 |
| PRPF4 | CDC26 | chr9_113369307_113370242 |
| LINC02614 | SLC41A3 | chr3_125912591_125912981 |
| FAM50A | PLXNA3 | chrX_154383823_154384184 |
| PCDHB16 | PCDHB11 | chr5_141040336_141041196 |
| STMP1 | SLC13A4 | chr7_135673224_135674002 |
| RMDN1 | WWP1 | chr8_86507398_86509382 |
| CPNE7 | LINC02166 | chr16_89581662_89583264 |
| LINC02701 | RP11-554A11 | chr11_69034632_69036450 |
| RP11-99J16__A | CAPN9 | chr1_230772802_230773110 |
| LMF1 | CEROX1 | chr16_963908_964405 |
| RP11-582E3 | MED21 | chr12_27222357_27222666 |
| SNX32 | CTSW | chr11_65989173_65989866 |
| SELENOO | HDAC10 | chr22_50219658_50220237 |
| HRAS | TMEM80 | chr11_605676_607411 |
| LRRC23 | C12orf57 | chr12_6880031_6881243 |
| CAPG | AC093162 | chr2_85407729_85411678 |
| PDHX | APIP | chr11_35005696_35006958 |
| NEK3 | ALG11 | chr13_51999168_52000097 |
| HCG4P3 | HCG9 | chr6_30097349_30098633 |
| XRCC3 | COA8 | chr14_103704405_103706431 |
| ASTE1 | NEK11 | chr3_130905182_130905717 |
| SLC35F5 | DDX11L2 | chr2_113895794_113896311 |
| KB-226F1 | AP000351 | chr22_23919167_23920601 |
| PDE6B-AS1 | SLC49A3 | chr4_680669_681509 |
| PRR4 | SMIM10L1 | chr12_11071427_11071793 |
| PRDX5 | PPP1R14B | chr11_64310715_64311218 |
| CAPN10 | GPR35 | chr2_240612327_240612823 |
| XCL1 | XCL2 | chr1_168534856_168535755 |
| MUC20P1 | MUC20 | chr3_195806218_195806508 |
| AC005082 | GPNMB | chr7_23246127_23248572 |
| AP000892 | RP11-109L13 | chr11_117225827_117226967 |
| HLA-DRB5 | HLA-DQB1 | chr6_32622554_32623136 |
| FLACC1 | CASP8 | chr2_201465519_201466392 |
| RP11-526I2 | LINS1 | chr15_100516399_100516827 |
| SNAI3-AS1 | RNF166 | chr16_88766117_88775626 |
| PGPEP1L | LUNAR1 | chr15_99018410_99018768 |
| ZNF337 | NANP | chr20_25548454_25549464 |
| ZNF232-AS1 | CAMTA2 | chr17_5226585_5227626 |
| ZNF302 | ZNF181 | chr19_34667136_34668083 |
| RAB29 | PM20D1 | chr1_205805646_205806386 |
| RNF39 | POLR1H | chr6_30097349_30098633 |
| OSGEPL1-AS1 | ORMDL1 | chr2_189639247_189640309 |
| ULK3 | MPI | chr15_74901863_74902826 |
| TUBG2 | CNTNAP1 | chr17_42666320_42670146 |
| FNTB | RAB15 | chr14_65096096_65097014 |
| MRPL19 | RP11-342K6 | chr2_75560738_75561335 |
| LYG1 | LIPT1 | chr2_99474758_99476472 |
| ALPK3 | NMB | chr15_84858301_84859870 |
| CTSW | SNX32 | chr11_65896772_65897096 |
| ACTR1B | ANKRD36B | chr2_97765892_97766437 |
| RP11-554A11 | IGHMBP2 | chr11_69034632_69036450 |
| EEF1AKMT3 | ATP23 | chr12_57886416_57886971 |
| SLC23A1 | DNAJC18 | chr5_139543198_139544031 |
| CCDC18 | DR1 | chr1_93322607_93326189 |
| IGHMBP2 | RP11-554A11 | chr11_69034632_69036450 |
| RP11-166O4 | STAG3L4 | chr7_67143645_67143895 |
| INTS4P1 | GTF2IP14 | chr7_65251945_65252713 |
| RP11-460N20 | INTS4P1 | chr7_65251945_65252713 |
| MICE | HCG4B | chr6_29648998_29650250 |
| SMPDL3B | XKR8 | chr1_27942802_27944630 |
| POLR1H | HCG4P3 | chr6_30091615_30092696 |
| TOMM7 | SNHG26 | chr7_22864979_22865523 |
| ZNF603P | ZSCAN26 | chr6_28334495_28337742 |
| PPDPF | FNDC11 | chr20_63530602_63531101 |
| LNCSRLR | PLOD2 | chr3_146152082_146154249 |
| RUFY2 | DNA2 | chr10_68348688_68349317 |
| RPL10P7 | CFAP91 | chr3_119659631_119661999 |
| PSORS1C2 | POU5F1 | chr6_31246281_31246849 |
| SNAPC4 | DNLZ | chr9_136493125_136494399 |
| SUOX | RPS26 | chr12_56084622_56088167 |
| CEP85 | SH3BGRL3 | chr1_26305645_26307823 |
| PROB1 | DNAJC18 | chr5_139438546_139440267 |
| ENTPD6 | ABHD12 | chr20_25384430_25384827 |
| WWP1 | RMDN1 | chr8_86507398_86509382 |
| RP11-326C3 | BET1L | chr11_267564_271662 |
| CTBS | SPATA1 | chr1_84618509_84621166 |
| DOC2A | YPEL3 | chr16_30084629_30085646 |
| HLA-H | POLR1H | chr6_29963963_29968759 |
| SLC7A9 | TDRD12 | chr19_32988243_32988941 |
| NAPEPLD | ARMC10 | chr7_103314370_103314903 |
| GPNMB | AC005082 | chr7_23334858_23335386 |
| SCNN1D | ANKRD65 | chr1_1264513_1264873 |
| LINC00933 | NMB | chr15_84724499_84725842 |
| THUMPD3 | THUMPD3-AS1 | chr3_9451379_9451643 |
| SLC35A1 | C6orf163 | chr6_87658860_87661487 |
| BTN3A1 | BTN3A2 | chr6_26532105_26533679 |
| HMGN2P19 | NUP133 | chr1_229582037_229582706 |
| DNAJC18 | PROB1 | chr5_139438546_139440267 |
| EML3 | EEF1G | chr11_62639512_62640028 |
| SPDYE5 | PMS2P3 | chr7_75564702_75565979 |
| RP11-44F14 | RBL2 | chr16_53510823_53511459 |
| DGCR5 | DGCR11 | chr22_19108756_19111028 |
| NMB | ALPK3 | chr15_84844572_84846175 |
| STPG3 | NELFB | chr9_137267757_137270336 |
| CICP14 | RP11-274B21 | chr7_128757673_128757910 |
| CHCHD2 | SUMF2 | chr7_56098210_56098901 |
| AMT | BSN-DT | chr3_49539125_49540748 |
| RNASEH2C | SNX32 | chr11_65686318_65686595 |
| IQGAP1 | CRTC3 | chr15_90556590_90563196 |
| TONSL-AS1 | TONSL | chr8_144422485_144423634 |
| RPL32P3 | IFT122 | chr3_129500902_129501314 |
| SETD4 | LINC01436 | chr21_36073668_36074166 |
| HLA-L | HCG18 | chr6_30322407_30322753 |
| ZNF586 | ZNF587 | chr19_57800918_57806967 |
| FAM3A | PLXNA3 | chrX_154383823_154384184 |
| TPSG1 | RP11-616M22 | chr16_1192303_1194266 |
| LINC02166 | CDK10 | chr16_89863194_89864881 |
| AP003068 | SLC22A20P | chr11_65378462_65379090 |
| SPSB2 | C12orf57 | chr12_6880031_6881243 |
| CTD-2248H3 | CKMT2 | chr5_81212704_81213651 |
| HLA-DPB2 | HSD17B8 | chr6_33017744_33020132 |
| MROH6 | NAPRT | chr8_143564685_143565574 |
| PCGF2 | CISD3 | chr17_38882791_38887755 |
| MED21 | RP11-582E3 | chr12_27220606_27220871 |
| CLDN15 | FIS1 | chr7_101243001_101245741 |
| RP11-274B21 | RP11-274B21 | chr7_128560920_128561369 |
| RP4-605O3 | COX14 | chr12_50314268_50314506 |
| ADSS1 | AKT1 | chr14_104774659_104776375 |
| TRIM52-AS1 | CTC-338M12 | chr5_181235002_181235353 |
| PSORS1C1 | CCHCR1 | chr6_31246281_31246849 |
| APOM | MICB | chr6_31840412_31840726 |
| ZNF192P1 | ZSCAN31 | chr6_28334495_28337742 |
| HLA-V | HCP5B | chr6_29963963_29968759 |
| C2orf74 | AHSA2P | chr2_61326996_61327233 |
| AP001625 | RSPH1 | chr21_42537451_42540553 |
| GIGYF1 | TFR2 | chr7_100885350_100885857 |
| MAMDC2-AS1 | SMC5 | chr9_70285032_70285600 |
| MXD3 | PRELID1 | chr5_177339050_177339888 |
| COL28A1 | MIOS-DT | chr7_7562332_7562782 |
| IDUA | SLC26A1 | chr4_927718_928088 |
| RP11-247A12 | CRAT | chr9_129163074_129165421 |
| ABHD12 | PYGB | chr20_25540202_25540884 |
| ASNSD1 | ORMDL1 | chr2_189641205_189641812 |
| PCMT1 | RAET1G | chr6_149847843_149848458 |
| ZNF500 | MGRN1 | chr16_4747077_4747761 |
| NDE1 | AF001548 | chr16_15775822_15777674 |
| RP11-274B21 | FAM71F2 | chr7_128560920_128561369 |
| HLA-C | XXbac-BPG248L24 | chr6_31246281_31246849 |
| TTC19 | ADORA2B | chr17_16170205_16170921 |
| CRIPT | RP11-536C12 | chr2_46563993_46564771 |
| PSORS1C2 | HCG27 | chr6_31246281_31246849 |
| ZNF738 | ZNF429 | chr19_21321954_21322700 |
| CTD-3199J23 | RP11-242D8 | chr17_43334044_43334985 |
| LINC00886 | LEKR1 | chr3_156751174_156751499 |
| ACYP2 | HMGB1P31 | chr2_54114581_54116390 |
| PDF | TMED6 | chr16_69395928_69398781 |
| METTL21EP | BIVM | chr13_102871856_102874238 |
| PRIMPOL | CENPU | chr4_184684498_184685093 |
| UBE2D3 | UBE2D3-AS1 | chr4_102878104_102878481 |
| CDIP1 | NMRAL1 | chr16_4528825_4530073 |
| WDR27 | ERMARD | chr6_169697660_169697920 |
| PAOX | SPRN | chr10_133388322_133390977 |
| RPAP1 | TYRO3 | chr15_41604050_41604851 |
| HSD17B1 | CNTNAP1 | chr17_42670251_42670862 |
| SNHG26 | TOMM7 | chr7_22867426_22867816 |
| CTD-2561J22 | CTD-2561J22 | chr19_21504710_21506711 |
| SUMF2 | CHCHD2 | chr7_56097280_56098083 |
| ZNF670 | ZNF124 | chr1_247054110_247055755 |
| MIR6891 | MICB | chr6_31411633_31411895 |
| RSPH1 | AP001625 | chr21_42545023_42545546 |
| GCC2 | GCC2-AS1 | chr2_108639646_108640762 |
| AHSA2P | C2orf74 | chr2_61327376_61327664 |
| QRICH1 | RP11-3B7 | chr3_49238064_49238557 |
| PSORS1C2 | CCHCR1 | chr6_31246281_31246849 |
| LRRC17 | FBXL13 | chr7_102991165_102993993 |
| RP11-98I9 | RP1-199J3 | chr6_99562916_99563472 |
| TAF1C | MBTPS1 | chr16_84200188_84200653 |
| PSORS1C2 | HLA-C | chr6_31246281_31246849 |
| PMS2P3 | SPDYE5 | chr7_75564702_75565979 |
| HRNR | FLG | chr1_152188765_152189601 |
| MRPS18AP1 | NME6 | chr3_48400955_48404954 |
| NELFB | FAM166A | chr9_137281674_137281932 |
| HCG18 | XXbac-BPG283O16 | chr6_30322407_30322753 |
| FAM166A | NELFB | chr9_137267757_137270336 |
| TNS2 | RP11-983P16 | chr12_53042334_53043529 |
| TAPBPL | VAMP1 | chr12_6547038_6548090 |
| STRN4 | FKRP | chr19_46753579_46754056 |
| VAMP1 | CD27-AS1 | chr12_6547038_6548090 |
| ZC3H3 | RP11-661A12 | chr8_143564685_143565574 |
| SCRN2 | LRRC46 | chr17_48001483_48003754 |
| RIC8A | SIRT3 | chr11_267564_271662 |
| SPATC1L | MCM3AP-AS1 | chr21_46294832_46298372 |
| PROC | MAP3K2-DT | chr2_127437772_127439216 |
| LRRC37A15P | UBE2D3-AS1 | chr4_102878104_102878481 |
| PRR4 | TAS2R64P | chr12_11071427_11071793 |
| HLA-DRB6 | HLA-DQB1 | chr6_32622554_32623136 |
| NOMO3 | PKD1P2 | chr16_16214206_16214948 |
| KIAA0753 | ALOX15P1 | chr17_6592539_6592933 |
| RP11-463O9 | MTHFSD | chr16_86585669_86586400 |
| MRPL34 | OCEL1 | chr19_17349219_17349889 |
| AP4M1 | TRIM4 | chr7_100221509_100222393 |
| RP11-507K2 | SPATA7 | chr14_88667636_88668235 |
| NME2 | NME1 | chr17_51298010_51299142 |
| RRP7BP | A4GALT | chr22_42622941_42625214 |
| NUDT19 | SLC7A9 | chr19_32705529_32705939 |
| LYNX1 | THEM6 | chr8_142756634_142757640 |
| HEXD | NARF | chr17_82495603_82498594 |
| SIRT3 | RIC8A | chr11_267564_271662 |
| RPL7L1 | PEX6 | chr6_42911432_42911884 |
| GOLGA6L9 | GOLGA6L10 | chr15_82613636_82614109 |
| MIOS-DT | COL28A1 | chr7_7562332_7562782 |
| CFAP44 | SIDT1 | chr3_113551730_113552714 |
| GTF2IP20 | SEPTIN7P13 | chr1_223841032_223841267 |
| HLA-A | HCG4P5 | chr6_30097349_30098633 |
| RPS6KB2 | ANKRD13D | chr11_67579149_67580488 |
| LYRM9 | NOS2 | chr17_27818380_27818758 |
| SUDS3 | RP11-131L12 | chr12_118418034_118419487 |
| SPOUT1 | PKN3 | chr9_128909876_128915071 |
| MIOS-DT | RP5-1159O4 | chr7_7562332_7562782 |
| ANAPC1P1 | ANAPC1P2 | chr2_86823714_86824290 |
| CTB-47B11 | NIPAL4 | chr5_157501606_157502047 |
| OBSCN-AS1 | IBA57 | chr1_228178410_228180075 |
| YPEL4 | TIMM10 | chr11_57644454_57645045 |
| RPL12 | SLC2A8 | chr9_127548252_127549201 |
| TFR2 | GIGYF1 | chr7_100678254_100681281 |
| CCDC163 | MUTYH | chr1_45678895_45680133 |
| CTD-2012J19 | RP11-43F13 | chr5_1567514_1568430 |
| ALOX15P1 | C17orf100 | chr17_6592539_6592933 |
| RP1-45C12 | RP1-127D3 | chr1_171191151_171191968 |
| AP003068 | DPF2 | chr11_65378462_65379090 |
| CTBP1-DT | UVSSA | chr4_1351647_1352074 |
| PCNT | YBEY | chr21_46393719_46394291 |
| IBA57 | OBSCN-AS1 | chr1_228178410_228180075 |
| COQ6 | PTGR2 | chr14_74064777_74065327 |
| RBL2 | RP11-44F14 | chr16_53511665_53512081 |
| MPI | RPP25 | chr15_75045657_75048774 |
| ZSCAN31 | ZKSCAN3 | chr6_28382912_28384280 |
| IVD | KNSTRN | chr15_40421669_40422184 |
| OR7E14P | PLEKHA7 | chr11_17197309_17197602 |
| TONSL-AS1 | CPSF1 | chr8_144422485_144423634 |
| NBPF2P | NBPF3 | chr1_21517469_21517944 |
| ALMS1P1 | TPRKB | chr2_73685743_73686090 |
| LINC01436 | SETD4 | chr21_36073668_36074166 |
| HCG9 | TRIM31-AS1 | chr6_30097349_30098633 |
| PMS2 | CCZ1 | chr7_6159454_6161144 |
| ACSM3 | ACSM1 | chr16_20734392_20735090 |
| FUT2 | RASIP1 | chr19_48775452_48776715 |
| NMT1 | DCAKD | chr17_45108919_45109842 |
| BAG5 | RP11-894P9 | chr14_103624602_103626726 |
| PRSS30P | LA16c-325D7 | chr16_2905820_2906892 |
| PTGR2 | COQ6 | chr14_73995744_73997598 |
| RP11-983P16 | TNS2 | chr12_53038047_53039799 |
| NARF | HEXD | chr17_82495603_82498594 |
| SMBD1P | MUC20 | chr3_195892189_195897762 |
| BANF1 | SNX32 | chr11_65989173_65989866 |
| HLA-DQB1 | HLA-DRB6 | chr6_32622554_32623136 |
| GOLGA6L9 | GOLGA2P10 | chr15_82613636_82614109 |
| RP11-507K2 | RP11-507K2 | chr14_88667636_88668235 |
| PPP1R13L | ERCC1 | chr19_45426490_45429637 |
| AC093162 | TGOLN2 | chr2_85412699_85415195 |
| ARL3 | BORCS7 | chr10_102739014_102739512 |
| HLA-K | HCG4P3 | chr6_30122290_30123759 |
| RNF166 | RP5-1142A6 | chr16_88766117_88775626 |
| RP5-1057I20 | RAPGEF3 | chr12_47779600_47780355 |
| PTGR2 | RP5-1021I20 | chr14_73995744_73997598 |
| ZNRD1ASP | HCG9 | chr6_30097349_30098633 |
| PCDHB11 | PCDHB18P | chr5_141084655_141085117 |
| RPS8 | DYNLT4 | chr1_44836238_44836482 |
| CTD-2561J22 | LINC00664 | chr19_21504710_21506711 |
| PNPLA7 | DPH7 | chr9_137556384_137556770 |
| FASTK | AGAP3 | chr7_151117151_151117446 |
| SMC5 | MAMDC2-AS1 | chr9_70285032_70285600 |
| ACSM1 | ACSM3 | chr16_20883198_20884679 |
| HLA-F | HCP5B | chr6_29648998_29650250 |
| RP11-387H17 | GSDMB | chr17_39949326_39949561 |
| RPA2 | XKR8 | chr1_27942802_27944630 |
| HLA-S | MICA | chr6_31246281_31246849 |
| ULK3 | RPP25 | chr15_74901863_74902826 |
| SPRN | SCART1 | chr10_133388322_133390977 |
| SLC41A1 | PM20D1 | chr1_205805646_205806386 |
| RP11-465B22 | C1orf159 | chr1_1156242_1161390 |
| RP11-661A12 | RP11-661A12 | chr8_143564685_143565574 |
| PES1 | SLC35E4 | chr22_30650388_30651228 |
| NELFB | STPG3-AS1 | chr9_137281674_137281932 |
| SIDT1 | CFAP44 | chr3_113557502_113558321 |
| SFTPD | PLAC9 | chr10_80170346_80173799 |
| HLA-A | HLA-K | chr6_30097349_30098633 |
| RRAS2 | COPB1 | chr11_14423579_14424183 |
| WDR6 | IHO1 | chr3_48939945_48940701 |
| HLA-DQB2 | HLA-DQB1 | chr6_32622554_32623136 |
| TAS2R64P | PRR4 | chr12_11071427_11071793 |
| ZNF500 | ROGDI | chr16_4747077_4747761 |
| MRPL34 | ANKLE1 | chr19_17349219_17349889 |
| POM121C | PMS2P3 | chr7_75564702_75565979 |
| NDUFB11 | RGN | chrX_47200824_47201583 |
| ARHGEF19 | CPLANE2 | chr1_16393277_16393763 |
| SNHG15 | TBRG4 | chr7_45026692_45028646 |
| LINC00957 | DBNL | chr7_44086933_44091195 |
| ZNF584 | SLC27A5 | chr19_58517753_58521091 |
| APOBEC3G | APOBEC3D | chr22_39116327_39116562 |
| GSTT2B | AP000351 | chr22_23919167_23920601 |
| HLA-C | XXbac-BPG181B23 | chr6_31246281_31246849 |
| ACOT4 | ACOT2 | chr14_73737021_73737388 |
| COX14 | RP4-605O3 | chr12_50135605_50136024 |
| AP001505 | LINC01547 | chr21_45029402_45031470 |
| PPP1R21 | GTF2A1L | chr2_48551243_48551746 |
| PPID | U3 | chr4_158808627_158812781 |
| ERCC3 | MAP3K2-DT | chr2_127437772_127439216 |
| SLC35E2A | RP1-140A9 | chr1_1789091_1789741 |
| ABCB8 | FASTK | chr7_151038424_151040544 |
| SH3BGRL3 | CEP85 | chr1_26321381_26321616 |
| DHX58 | RAB5C | chr17_42142059_42143943 |
| CDK10 | SPATA2L | chr16_89853172_89853407 |
| LINC01529 | U2AF1L4 | chr19_35733619_35734042 |
| BSN-DT | AMT | chr3_49665318_49667004 |
| SERBP1P3 | RFT1 | chr3_53133894_53135294 |
| SLC9A3-AS1 | EXOC3 | chr5_671472_671740 |
| STIM2 | STIM2-AS1 | chr4_26907405_26908361 |
| PRC1-AS1 | RCCD1 | chr15_90988318_90988769 |
| CECR7 | AC006946 | chr22_17062787_17064779 |
| PSORS1C3 | CCHCR1 | chr6_31246281_31246849 |
| RP11-20I20 | UVSSA | chr4_1175119_1175836 |
| CYP2D6 | CYP2D8P | chr22_42218119_42218456 |
| LINC00957 | POLM | chr7_44086933_44091195 |
| APTR | PTPN12 | chr7_77735100_77736034 |
| TRMT61B | PPP1CB | chr2_29013815_29017658 |
| LYNX1 | LY6D | chr8_142756634_142757640 |
| SKA2 | TRIM37 | chr17_59002228_59002660 |
| SURF1 | SURF6 | chr9_133469011_133472196 |
| ATAD3C | MRPL20 | chr1_1440445_1441552 |
| HLA-V | ZFP57 | chr6_29963963_29968759 |
| B4GALT7 | TMED9 | chr5_177677654_177678000 |
| C3orf86 | ZNF660 | chr3_44355093_44356643 |
| RP11-331F4 | CFDP1 | chr16_75262182_75263099 |
| TAPBPL | CD27-AS1 | chr12_6547038_6548090 |
| PLXNA3 | FAM50A | chrX_154383823_154384184 |
| TRBC2 | PRSS2 | chr7_142807162_142808816 |
| IQCD | RITA1 | chr12_113258646_113260907 |
| XXbac-BPG299F13 | MICA | chr6_31246281_31246849 |
| ACBD4 | DCAKD | chr17_45122072_45126019 |
| S100A10 | THEM4 | chr1_151982654_151982910 |
| SLC35F5 | RPL23AP7 | chr2_113895794_113896311 |
| HLA-DQA1 | HLA-DOB | chr6_32622554_32623136 |
| RP11-552M11 | UBE2FP3 | chr1_111406599_111406843 |
| CAMTA2 | ZNF232-AS1 | chr17_5096976_5097829 |
| TRMU | PKDREJ | chr22_46334922_46336940 |
| RP11-345P4 | SLC35E2A | chr1_1662684_1663406 |
| RP11-507K2 | PTPN21 | chr14_88667636_88668235 |
| LIG3 | CCT6B | chr17_35062613_35065197 |
| ANKLE2 | GOLGA3 | chr12_132858961_132859337 |
| SNX32 | BANF1 | chr11_65989173_65989866 |
| RPL23AP7 | SLC35F5 | chr2_113818580_113819007 |
| PRSS12 | SNHG8 | chr4_118345884_118347235 |
| RP11-532F6 | EXOC2 | chr6_776212_776464 |
| RP11-17E13 | CRYZ | chr1_74713046_74713723 |
| RPP25 | MPI | chr15_75151402_75151705 |
| TAZ | FAM50A | chrX_154357529_154357811 |
| CDC26 | PRPF4 | chr9_113369307_113370242 |
| IHO1 | WDR6 | chr3_49296765_49297702 |
| SAT2 | FXR2 | chr17_7650777_7651042 |
| PPDPF | SRMS | chr20_63530602_63531101 |
| NMB | RP11-182J1 | chr15_84844572_84846175 |
| IFRD2 | HYAL3 | chr3_50179166_50180612 |
| STIM2-AS1 | STIM2 | chr4_27009526_27009770 |
| ERAP1 | CTD-2260A17 | chr5_96880363_96881808 |
| RCC1L | GTF2IP1 | chr7_75054419_75054686 |
| AGAP5 | FUT11 | chr10_73848657_73854871 |
| PCAT19 | LINC01480 | chr19_41480113_41480789 |
| RP1-140A9 | CALML6 | chr1_1908215_1910200 |
| SNHG29 | LRRC75A | chr17_16485817_16487145 |
| CD151 | TSPAN4 | chr11_849912_850249 |
| RP1-40E16 | NQO2 | chr6_2940546_2940885 |
| FAM160B2 | NUDT18 | chr8_22117695_22117930 |
| SULT1A2 | SGF29 | chr16_28609119_28610955 |
| AC016747 | RP11-493E12 | chr2_61170554_61170833 |
| FAM153A | B4GALT7 | chr5_177614802_177615050 |
| RP11-298I3 | PSMB5 | chr14_22951235_22951945 |
| NOMO3 | ABCC6 | chr16_16214206_16214948 |
| UBXN6 | CTB-50L17 | chr19_4396555_4396994 |
| SETD2 | NBEAL2 | chr3_47140765_47141452 |
| GCC2-AS1 | GCC2 | chr2_108661571_108662955 |
| MRPL35 | IMMT | chr2_86302165_86302400 |
| HCG4P3 | POLR1H | chr6_30097349_30098633 |
| CICP14 | FAM71F2 | chr7_128757673_128757910 |
| RGS11 | LA16c-OS12 | chr16_325706_328986 |
| NBR2 | RP11-242D8 | chr17_43314726_43317127 |
| NSUN2 | LINC01018 | chr5_6598717_6598952 |
| NDUFB2 | ADCK2 | chr7_140656063_140657451 |
| INO80E | YPEL3 | chr16_30166478_30166716 |
| CCDC169 | SOHLH2 | chr13_36417483_36418025 |
| KANSL1-AS1 | NSFP1 | chr17_46284944_46285558 |
| MRPL35 | POLR1A | chr2_86302165_86302400 |
| P4HTM | QRICH1 | chr3_48939945_48940701 |
| ANKRD36B | ACTR1B | chr2_97765892_97766437 |
| DIS3L | TIPIN | chr15_66472069_66472769 |
| MICB | LY6G5B | chr6_31562996_31563276 |
| HLA-K | RPL23AP1 | chr6_30122290_30123759 |
| SIRT3 | RP11-326C3 | chr11_267564_271662 |
| TIPARP-AS1 | LINC00886 | chr3_156725990_156726622 |
| PSORS1C2 | XXbac-BPG299F13 | chr6_31246281_31246849 |
| RP11-94C24 | MYCBPAP | chr17_50351611_50352527 |
| CATSPERG | PPP1R14A | chr19_38395880_38396281 |
| PCDHB16 | PCDHB8 | chr5_141040336_141041196 |
| ZNF740 | SOAT2 | chr12_53200409_53201020 |
| ZNF548 | ZNF749 | chr19_57338104_57338898 |
| VPS11 | HMBS | chr11_119192046_119192320 |
| SPATA1 | CTBS | chr1_84618509_84621166 |
| CDK3 | EVPL | chr17_76004198_76005952 |
| MIF | KB-226F1 | chr22_23919167_23920601 |
| THEM6 | JRK | chr8_142756634_142757640 |
| EDEM2 | MYH7B | chr20_35087843_35088418 |
| GSTM5 | GSTM3 | chr1_109716503_109716817 |
| DDX19A-DT | COG4 | chr16_70477365_70477600 |
| AP000351 | KB-226F1 | chr22_23919167_23920601 |
| PARD6G-AS1 | AC139100 | chr18_79987765_79989516 |
| LRRC46 | SCRN2 | chr17_48025359_48026156 |
| ZNF184 | ZNF391 | chr6_27478112_27480195 |
| UBE2D3-AS1 | UBE2D3 | chr4_103011296_103013239 |
| DBF4B | EFTUD2 | chr17_44871896_44872670 |
| HNRNPA1P49 | SFT2D1 | chr6_166330549_166330827 |
| BTNL8 | BTNL9 | chr5_180889392_180889925 |
| TNS2 | EIF4B | chr12_53042334_53043529 |
| BCO2 | IL18 | chr11_112160082_112160490 |
| MICB | HLA-S | chr6_31562996_31563276 |
| TUFM | SH2B1 | chr16_28923077_28926410 |
| TBKBP1 | MRPL45P2 | chr17_47708112_47709593 |
| XXbac-BPG299F13 | XXbac-BPG181B23 | chr6_31246281_31246849 |
| C17orf100 | ALOX15P1 | chr17_6574685_6576100 |
| GSTM3 | GSTM5 | chr1_109705668_109706153 |
| STRN4 | PRKD2 | chr19_46753579_46754056 |
| CTD-2349P21 | SUZ12P1 | chr17_30840386_30841399 |
| PCMT1 | RP11-350J20 | chr6_149847843_149848458 |
| ZNF582-DT | ZNF667-AS1 | chr19_56403200_56404818 |
| RP11-461A8 | CLUAP1 | chr16_3695739_3697017 |
| HLA-C | POU5F1 | chr6_31246281_31246849 |
| TRAPPC4 | RPS25 | chr11_119024280_119025896 |
| ZSCAN26 | ZSCAN31 | chr6_28382912_28384280 |
| CTD-2619J13 | SLC27A5 | chr19_58580254_58581810 |
| NAPRT | MROH6 | chr8_143564685_143565574 |
| ALDH9A1 | TMCO1-AS1 | chr1_165647452_165647784 |
| PIGP | TTC3 | chr21_37259656_37259960 |
| ZNF396 | ZNF24 | chr18_35247021_35247418 |
| C1orf174 | DFFB | chr1_3903422_3903768 |
| PRKD2 | STRN4 | chr19_46761252_46761771 |
| OSGEPL1-AS1 | PMS1 | chr2_189639247_189640309 |
| DR1 | CCDC18 | chr1_93361638_93362286 |
| PBLD | DNA2 | chr10_68305390_68307477 |
| CCDC32 | BAHD1 | chr15_40744056_40744412 |
| AL022393 | ZSCAN31 | chr6_28334495_28337742 |
| TGOLN2 | AC093162 | chr2_85412699_85415195 |
| SMTNL1 | TIMM10 | chr11_57679527_57680931 |
| RP5-1021I20 | PTGR2 | chr14_73913694_73913969 |
| MYH7B | EDEM2 | chr20_35151979_35152523 |
| GRB7 | PGAP3 | chr17_39769626_39769986 |
| MIR6891 | PSORS1C3 | chr6_31411633_31411895 |
| MMP24OS | RP4-614O4 | chr20_35269095_35269756 |
| DGKQ | SLC26A1 | chr4_979199_979552 |
| SNHG8 | PRSS12 | chr4_118345884_118347235 |
| SEC1P | FUT2 | chr19_48728903_48729269 |
| ZSCAN23 | ZSCAN26 | chr6_28382912_28384280 |
| MIR6891 | HLA-B | chr6_31411633_31411895 |
| CECR7 | IL17RA | chr22_17062787_17064779 |
| PRAM1 | MARCHF2 | chr19_8517577_8518690 |
| TBRG4 | NACAD | chr7_45026692_45028646 |
| MICA | HLA-S | chr6_31562996_31563276 |
| HLA-L | XXbac-BPG283O16 | chr6_30322407_30322753 |
| HLA-DQB1-AS1 | HLA-DQA2 | chr6_32622554_32623136 |
| AP001469 | SPATC1L | chr21_46188528_46189869 |
| FAAP24 | SLC7A9 | chr19_32976426_32977725 |
| LA16c-390E6 | CCDC154 | chr16_1343311_1343948 |
| PHF10 | WDR27 | chr6_169654227_169655591 |
| TEX261 | ANKRD53 | chr2_70984915_70985624 |
| RP11-242D8 | CTD-3199J23 | chr17_43366850_43370773 |
| TNXA | RNF5 | chr6_32058086_32058452 |
| SDHAP3 | CTD-2245E15 | chr5_1582731_1583963 |
| RP11-326C3 | SIRT3 | chr11_267564_271662 |
| BTBD19 | RPS8 | chr1_44833878_44834113 |
| RP11-333E1 | ZNF232-AS1 | chr17_5226585_5227626 |
| RP11-147I3 | XRRA1 | chr11_74909239_74909765 |
| ZFP57 | IFITM4P | chr6_29648998_29650250 |
| RNFT1 | RPS6KB1 | chr17_59832824_59833466 |
| AGER | RNF5 | chr6_32244418_32245347 |
| PPP1R14A | CATSPERG | chr19_38273461_38275150 |
| LYNX1 | PSCA | chr8_142756634_142757640 |

**Table S2.** Class A orphan GPCRs in GC tumors

| Class A orphan GPCRs | GC-Expression^$^ | GC-Survival^#^ |
| --- | --- | --- |
| CMKLR1 | 1.57797E-05 | NA |
| FFAR4 | >0.05 | NA |
| GPR1 | 0.10112 | NA |
| GPR101 | >0.05 | NA |
| GPR12 | 0.0028391 | NA |
| GPR132 | 0.0001568 | NA |
| GPR135 | 0.0169231 | NA |
| GPR139 | 0.21066 | NA |
| GPR141 | 2.8055E-10 | NA |
| GPR142 | 0.87242 | NA |
| GPR146 | 0.000156933 | NA |
| GPR148 | 0.34842 | NA |
| GPR149 | 0.68368 | NA |
| GPR15 | 0.086297 | NA |
| GPR150 | 7.4211E-09 | NA |
| GPR151 | 0.23306 | NA |
| GPR152 | 1.2644E-10 | NA |
| GPR153 | 0.0101812 | NA |
| GPR160 | 1.0604E-09 | 0.71 |
| GPR161 | 0.190239 | NA |
| GPR162 | 0.5371 | NA |
| GPR171 | 0.0171758 | 0.9 |
| GPR173 | 0.0039571 | 0.093 |
| GPR176 | <1E-12 | 0.079 |
| GPR182 | 0.043497 | 0.55 |
| GPR183 | 0.090543 | NA |
| GPR19 | 1.62448E-12 | 0.91 |
| GPR20 | 0.0069757 | 0.32 |
| GPR21 | 0.0036989 | 0.37 |
| GPR22 | 0.052256 | NA |
| GPR25 | 0.0151836 | 0.22 |
| GPR26 | 0.045939 | 0.015 |
| GPR27 | 0.0154024 | 0.52 |
| GPR3 | 0.00042453 | 0.94 |
| GPR31 | 0.191623 | NA |
| GPR32 | 0.46486 | NA |
| GPR32P1 | >0.05 | NA |
| GPR33 | >0.05 | NA |
| GPR34 | 0.00043592 | 0.0015 |
| **GPR35** | **0.0088584** | **4.3e-08** |
| GPR37 | 0.89602 | NA |
| GPR37L1 | 0.64632 | NA |
| GPR39 | 0.00153843 | 0.91 |
| GPR4 | 1.24345E-14 | 0.24 |
| GPR42 | >0.05 | NA |
| GPR45 | 2.3206E-05 | 0.84 |
| GPR52 | 0.097798 | NA |
| GPR55 | 0.180085 | NA |
| GPR61 | 0.63234 | NA |
| GPR62 | 0.021265 | 0.041 |
| GPR63 | 3.0321E-11 | 0.81 |
| GPR68 | 0.000175125 | 0.74 |
| GPR75 | 0.0028761 | 0.44 |
| GPR78 | 0.199979 | NA |
| GPR82 | 0.112635 | NA |
| GPR83 | 0.24502 | NA |
| GPR84 | 1.62437E-12 | 0.59 |
| GPR85 | 1.62448E-12 | 0.0043 |
| GPR87 | 0.106421 | NA |
| GPR88 | 0.44274 | NA |
| LGR4 | 4.44089E-16 | 0.23 |
| LGR5 | 1.62437E-12 | 0.41 |
| LGR6 | 2.1534E-08 | 0.027 |
| MAS1 | 0.00038332 | NA |
| MAS1L | 0.064598 | NA |
| MRGPRD | 0.037941 | 0.038 |
| MRGPRE | 0.26524 | NA |
| MRGPRF | 0.0041177 | 0.028 |
| MRGPRG | >0.05 | NA |
| MRGPRX1 | >0.05 | NA |
| MRGPRX2 | 0.0024216 | 0.055 |
| MRGPRX3 | 0.39274 | NA |
| MRGPRX4 | 3.1242E-07 | NA |
| PTGDR2 | p<0.05 | 0.8 |

^$^ represents p value for difference between gene expression normal and matched tumor tissue; ^#^ represents p value for overall survival of significance difference expression gene in GC patients. NA indicates no data or not applicable.

**Table S3.** ERR events in kidney

| **Gene-CP** | **Gene-AP** | **enhancer (hg38)** |
| --- | --- | --- |
| AP000350 | KB-226F1 | chr22_23993841_23994022 |
| AP000350 | GSTT2 | chr22_23993841_23994022 |
| AP000350 | KB-226F1 | chr22_23913403_23914032 |
| C2orf74 | AHSA2P | chr2_61177443_61178898 |
| CCT6P3 | INTS4P1 | chr7_64997557_65000755 |
| EEF1DP4 | ERV3-1 | chr7_64945249_64946968 |
| EEF1DP4 | CCT6P3 | chr7_64945249_64946968 |
| ERV3-1 | INTS4P1 | chr7_64997557_65000755 |
| GPNMB | KLHL7-DT | chr7_23181471_23182703 |
| GTF2IP14 | INTS4P1 | chr7_65269232_65270105 |
| IGHMBP2 | LINC02701 | chr11_68857586_68858348 |
| INTS4P1 | GTF2IP14 | chr7_65269232_65270105 |
| KB-226F1 | AP000351 | chr22_23993841_23994022 |
| KLHL7-DT | GPNMB | chr7_23299356_23300113 |
| LINC02701 | IGHMBP2 | chr11_68857586_68858348 |
| MIF-AS1 | KB-226F1 | chr22_23993841_23994022 |
| PEX6 | GNMT | chr6_42970855_42972500 |
| PSORS1C3 | CCHCR1 | chr6_31203408_31204378 |
| XRRA1 | RP11-147I3 | chr11_75029694_75032674 |
